# A nonalcoholic fatty liver disease model in human induced pluripotent stem cell-derived hepatocytes, created by endoplasmic reticulum stress-induced steatosis

**DOI:** 10.1242/dmm.033530

**Published:** 2018-09-25

**Authors:** Maddalena Parafati, R. Jason Kirby, Sepideh Khorasanizadeh, Fraydoon Rastinejad, Siobhan Malany

**Affiliations:** 1 Translational Biology, Conrad Prebys Center for Chemical Genomics, Orlando, FL 32827, USA; 2 Center for Metabolic Origins of Disease, Sanford Burham Prebys Medical Discovery Institute, 6400 Sanger Rd, Orlando, FL 32827, USA

**Keywords:** Induced pluripotent stem cell-derived hepatocytes, NAFLD *in vitro* model, ER stress, Phenotypic high-content analysis, Lipid accumulation, RNA sequencing

## Abstract

Hepatic steatosis, a reversible state of metabolic dysregulation, can promote the onset of nonalcoholic steatohepatitis (NASH), and its transition is thought to be critical in disease evolution. The association between endoplasmic reticulum (ER) stress response and hepatocyte metabolism disorders prompted us to characterize ER stress-induced hepatic metabolic dysfunction in human induced pluripotent stem cell-derived hepatocytes (hiPSC-Hep), to explore regulatory pathways and validate a phenotypic *in vitro* model for progression of liver steatosis. We treated hiPSC-Hep with a ratio of unsaturated and saturated fatty acids in the presence of an inducer of ER stress to synergistically promote triglyceride accumulation and dysregulate lipid metabolism. We monitored lipid accumulation by high-content imaging and measured gene regulation by RNA sequencing and reverse transcription quantitative PCR analyses. Our results show that ER stress potentiated intracellular lipid accumulation by 5-fold in hiPSC-Hep in the absence of apoptosis. Transcriptome pathway analysis identified ER stress pathways as the most significantly dysregulated of all pathways affected. Obeticholic acid dose dependently inhibited lipid accumulation and modulated gene expression downstream of the farnesoid X receptor. We were able to identify modulation of hepatic markers and gene pathways known to be involved in steatosis and nonalcoholic fatty liver disease (NAFLD), in support of a hiPSC-Hep disease model that is relevant to clinical data for human NASH. Our results show that the model can serve as a translational discovery platform for the understanding of molecular pathways involved in NAFLD, and can facilitate the identification of novel therapeutic molecules based on high-throughput screening strategies.

## Abstract (precis)

**Summary:** Our study demonstrates expanded use of human induced pluripotent stem cell-derived hepatocytes for molecular studies and drug screening, to evaluate new therapeutics with an antisteatotic mechanism of action for nonalcoholic fatty liver disease.

## INTRODUCTION

Hepatic steatosis, the first step in the progression of nonalcoholic fatty liver disease (NAFLD), characterized by excessive intracellular lipid accumulation in the form of cytosolic lipid droplets, can progress to nonalcoholic steatohepatitis (NASH) and hepatocellular injury (Bedossa, 2017; Vernon et al., 2011). In the western society, NAFLD affects ∼30% of the general population and 70-95% of individuals with type 2 diabetes and obesity (Bellentani, 2017). Sedentary lifestyle and high-fat diet contribute to the accumulation of fat as cytosolic lipid droplets, mainly in the form of triacylglycerols (TAGs) in the hepatocytes of NAFLD patients; and, if accumulation exceeds the liver metabolic capacity, steatotic hepatocytes become more vulnerable to various insults in progressive NAFLD (Veteläinen et al., 2007). Although metabolic syndrome plays a major role in most patients with NAFLD, a significant patient population is lean with few metabolic conditions (Ahmed, 2015). Molecular mechanisms related to genetic background in NAFLD progression of steatosis to NASH are complex and not completely understood. There are currently no US Food and Drug Administration (FDA)-approved medicines to treat NAFLD or NASH in humans (Filozof et al., 2015; Hegade et al., 2016).

Because accumulating evidence links the endoplasmic reticulum (ER) stress response in both exacerbating liver steatosis and NAFLD progression (Lake et al., 2014; Gentile et al., 2011), we decided to target ER stress to increase steatosis. ER stress signaling pathways, notably the unfolded protein response (UPR) pathway, are triggered when high levels of saturated fatty acids (FAs) and misfolded proteins alter ER homeostasis, creating a lipotoxic environment in the liver upon NAFLD progression (Fuchs and Sanyal, 2012; Leamy et al., 2013). Disruption of ER homeostasis has been observed in the liver tissues of humans with NAFLD and/or obesity (Lake et al., 2014; Puri et al., 2008; Gregor et al., 2009). If homeostasis is not restored by activating UPR recovery pathways, improper responses to ER stress trigger hepatic fat accumulation and inflammation (Lee, 2012), insulin resistance (Kim et al., 2015; Ozcan et al., 2004) and apoptosis, which are the hallmarks of NAFLD (Zhang et al., 2014).

In parallel with ER stress-induced steatosis, intracellular hepatic lipids can accumulate as a result of decreased FA oxidation and TAG-rich very-low-density lipoprotein (VLDL) secretion, as well as increased uptake of circulating FA derived from the diet or synthesized *de novo* (Koo, 2013). In fact, ER stress could be a driver of VLDL receptor expression, leading to hepatic steatosis by increasing VLDL intracellular levels (Jo et al., 2013). Studies in humans revealed the importance of *de novo* lipogenesis in the excessive hepatic accumulation of TAG, contributing to about a quarter of liver lipids in patients affected by NAFLD (Donnelly et al., 2005; Lambert et al., 2014).

There is a necessity for *in vitro* models for drug discovery and development to recapitulate cellular properties of human NAFLD to discover new treatment strategies. Primary cultures of human hepatocytes represent substantial limitations that include de-differentiation, lack of precise availability and variable proliferation, which result in polymorphism in metabolic markers and gene expression; thus, are not suitable for use in drug discovery (Gerets et al., 2012; Zeilinger et al., 2016). Human induced pluripotent stem cell (hiPSC)-derived hepatocytes (hiPSC-Hep) generate mature hepatocytes in culture, with morphological and functional characteristics comparable to those of human primary hepatocytes (Lu et al., 2015; Mann, 2015; Takebe et al., 2014). The hiPSC-Hep display uniform quality and are available in high quantity for drug screening by phenotypic high-throughput imaging approaches (Avior et al., 2016; Sirenko et al., 2014, 2016). We describe the development and validation of a model of hepatic steatosis in functional hiPSC-Hep co-treated with FA and the ER stressor thapsigargin (TG). The accumulation of TAG, as well as gene expression alterations concerning *de novo* lipogenesis, FA and lipid metabolism, were measured by a combination of high-content analysis and transcriptomics. Obeticholic acid (OCA), a clinically advanced therapeutic and farnesoid X receptor (FXR) agonist, was used to validate our model by investigating functional crosstalk between FXR pathway activation and ER stress-induced signaling and lipid metabolism dysregulation. Our hiPSC-Hep disease phenotype exhibited metabolic changes characteristic of steatosis associated with NAFLD. The present NAFLD model permits interrogation of molecular pathways involved in disease progression and the discovery of new therapeutics by high-throughput screening technologies.

## RESULTS

### hiPSC-Hep present similar morphological and functional characteristics to primary hepatocytes

To highlight the advantage of using hiPSC-Hep for phenotypic analysis and pharmacological studies, we monitored morphological changes by high-content imaging (Fig. S1). By day 7 post-thaw, cells adopted a typical flat and polygonal shape and were occasionally bi-nucleated (Fig. S1A). The supernatant from cultured hiPSC-Hep at the end of the 9-day maturation cycle analyzed by enzyme-linked immunosorbent assay (ELISA) showed physiological levels of secreted albumin and urea (Fig. S1B). High-content imaging revealed that the hiPSC-Hep exhibit key hepatic characteristics, such as glycogen storage, bile canaliculi function, and albumin and lipid accumulation, similar to those exhibited by primary hepatocytes, as previously described in the literature (Mann et al., 2013; Lu et al., 2015; Sirenko et al., 2014).

To investigate the hepatic maturation state of hiPSC-Hep during time in culture, we examined the relative expression of common genes using reverse transcription quantitative PCR (RT-qPCR) analyses, and compared the results with expression data from primary hepatocytes seeded for 24 h and 48 h in parallel. After 48 h in culture, the primary hepatocytes decline rapidly, limiting our ability to directly compare gene levels after 9 days in culture with the hiPSC-Hep. Nonetheless, gene expression profiling revealed that key pluripotency markers including SR-related HMG-box 17 (*SOX17*) and POU class 5 homeobox 1 (*POU5F1*) displayed specific patterns of expression during *in vitro* maturation in hiPSC-Hep. Genes showing differences in expression patterns included alpha-fetoprotein (*AFP*), which is typically expressed in hepatocyte-like cultures but not in adult primary hepatocytes (Chen et al., 2012) (Fig. S2A-C). However, the hiPSC-Hep do express liver-related cytokeratin 8 (*KT8*), which is typically expressed by mature hepatocytes (Fig. S2D).

In addition, the generation of functional hepatocyte-like cells from hiPSCs was confirmed by the expression of liver-related genes, including hepatocyte nuclear factor 4 alpha (*HNF4A*), tryptophan 2,3-dioxygenase (*TDO2*), albumin (*ALB*) and cadherin-1 (*CDH1*) (Fig. S2E-H). The late-stage differentiation marker tyrosine aminotransferase (*TAT*) was, however, not expressed in hiPSC-Hep (Fig. S2I). We also measured the expression of genes encoding cytochrome P450 enzymes in hiPSC-Hep (Fig. S3A), observing a similar level of expression compared with primary hepatocytes for *CYP3A4* and *CYP2C9*, lower levels of *CYP2C19* (Fig. S3B-D), and high levels of phase II, histamine N-methyltransferase (*HNMT*) and phase III, ATP-binding cassette, sub-family B member 11 (*ABCB11*) (Fig. S3E,F), as well as increasing – but lower – levels of members of the organic acid transporter (SLC22A) gene family.

Our gene expression profiling confirm that hiPSC-Hep are fully differentiated and, while displaying fetal characteristics such as high *AFP* and low *TAT* levels, resemble the hepatic morphology, phenotype and functionality of adult primary hepatocytes. Although cytochrome P450 activities in these cells are in the low-to-average range overall, compared with primary cells as described (Lu et al., 2015), hiPSC-Hep show good prediction of drug-induced hepatotoxicity and are more sensitive relative to other hepatocyte sources (Kang et al., 2016; Kim et al., 2017; Takayama et al., 2014). The advantages to using hiPSC-Hep in drug discovery include their consistency in culture, unlimited availability, and automated and cost-effective scale up. Primary hepatocytes, on the other hand, exhibit variability and dedifferentiation in culture after 48 h, limiting their use as a throughput drug discovery platform (Fraczek et al., 2013). New engineering approaches using these hiPSC-Hep have shown enhanced cytochrome P450 activities and hepatocellular function (Sirenko et al., 2014; Berger et al., 2015). In addition, hiPSC-Hep derived from multiple donors, both healthy and diseased, have great potential to provide powerful pharmacological profiling platforms to highlight donor and patient-specific responses to drug candidates. This study is focused on incorporating hiPSC-Hep in a drug discovery platform to model signaling pathways most likely contributing to steatosis progression and related to NAFLD, as an initial approach to drug screening to be enhanced in future studies using co-cultures and patient-specific derived cells.

### TG potentiates FA-induced TAG accumulation in hiPSC-Hep

**Fig. 1.**
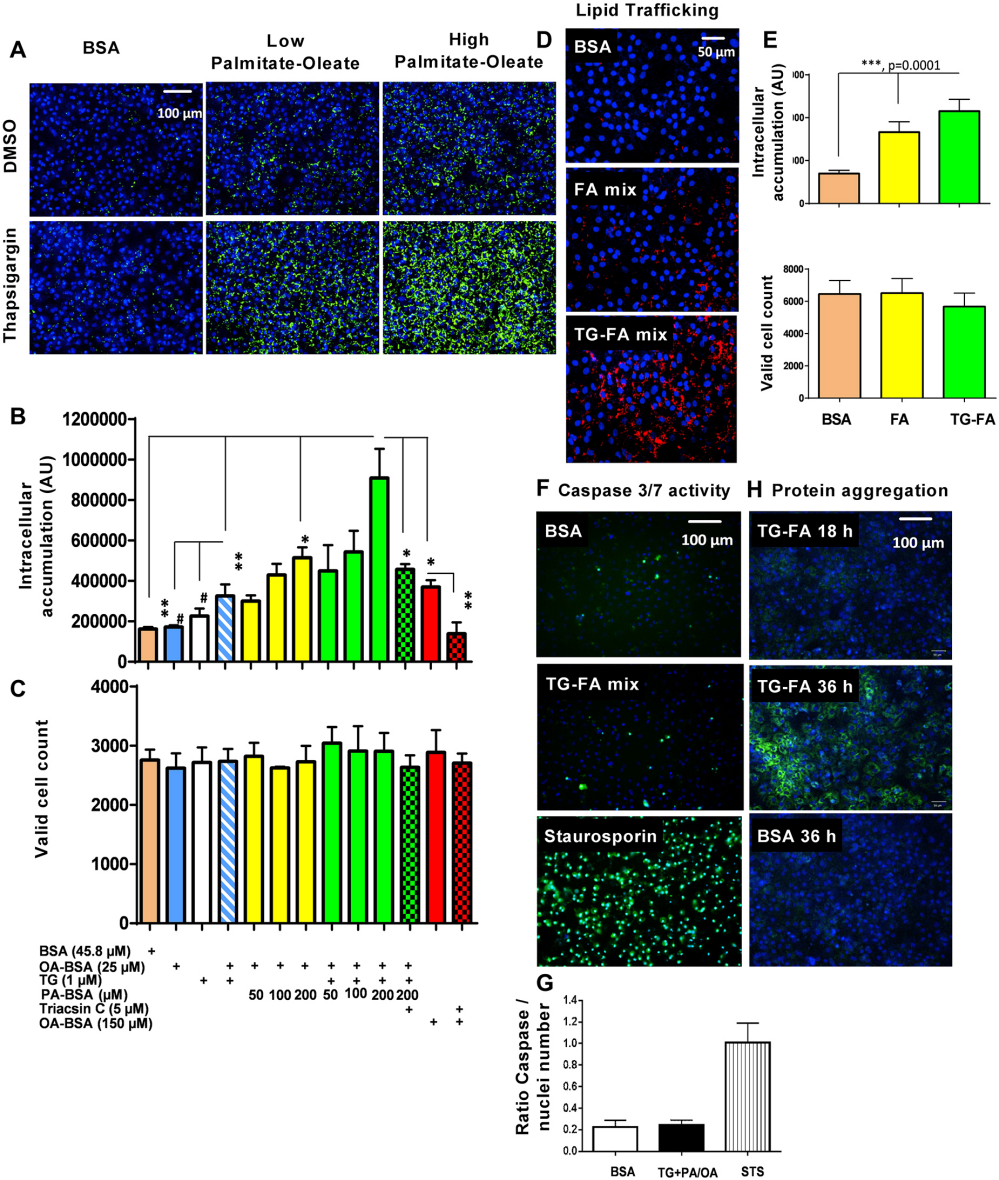
**Development of steatotic phenotype in hiPSC-Hep.** (A) BODIPY 493/503 staining of neutral lipid droplet (green) induced by co-treating cells with 50 (low) or 200 (high) µM PA and 25 µM OA with or without thapsigargin (TG) for 18 h compared with BSA-treated cells; nuclei were stained with Hoechst dye (blue). Scale bar: 100 µm. (B,C) Quantification of immunofluorescence in A in the BODIPY channel for lipid accumulation (B) and nuclear channel for number of valid nuclei (C). Data are mean±s.d. for three experimental determinations, where each determination includes the average of three wells and seven fields per well. *P≤0.01; **P≤0.006; ^#^*P*≤0.03. (D) 10 µM fluorescent fatty acid (FA) C_12_ analog uptake and accumulation in the BODIPY-FA 558/568 channel (red) in the presence of BSA, FA mix and TG-FA cocktail (TG-FA mix) treatments. Scale bar: 50 µm. (E) Quantification of fluorescent FA analog in BODIPY 558/568 and nuclear channels. (F) Evaluation of caspase 3/7 enzyme activation by confocal live imaging of hiPSC-Hep treated with BSA, TG-FA cocktail or staurosporin control for 18 h in the presence of CellEvent dye (green) and Hoechst (blue). Scale bar: 100 µm. (G) Quantification of caspase 3/7-positive hiPSC-Hep. Data are mean±s.d for two experimental determinations, where each determination includes the average of three wells and seven fields per well. (H) Evaluation of unfolded protein accumulation by confocal live imaging of hiPSC-Hep co-treated with 10 µM Thioflavin T and TG-FA cocktail and captured at 18 h and 36 h. Images of cells treated with BSA were used as a negative control. Scale bar: 100 µm.

Palmitic acid (PA) and oleic acid (OA) are the most abundant dietary long-chain FAs in liver triacylglycerides in both normal subjects and patients with NAFLD (Araya et al., 2004). Co-supplementation of saturated and monounsaturated FAs induces more steatosis compared with monounsaturated supplementation only (De Gottardi et al., 2007; Gómez-Lechón et al., 2007), and inhibits the toxicity of saturated FA channeling to TAG stores (Listenberger et al., 2003). We developed a multiparametric high-content 384w platform to induce FA uptake and triacylglyceride synthesis after exposure to OA in the presence of varying concentrations of PA to mimic nutrient overload (Fig. 1A). To quantify lipid droplets, we applied an algorithm to detect integrated spot signal of boron-dipyrromethene (BODIPY)-stained TAG in the whole cell region and monitor cell health in the nuclear region. PA-induced lipid droplets accumulated dose dependently in the presence of 25 µM OA up to 200 µM, the highest concentration tested that produced spot intensity 3.2-fold over bovine serum albumin (BSA)-treated cells without effect on valid cell count (Fig. 1B,C, yellow bars).

To test whether ER stress contributes to a more steatotic phenotype, cells were exposed to TG, a noncompetitive inhibitor of the sarcoplasmic reticulum/ER Ca^2+^ ATPase known to cause dysregulation of the UPR (Lytton, 1991; Achard and Laybutt, 2012). TG treatment alone did not cause significant lipid droplet accumulation at 1 µM but increased levels in the presence of 25 µM OA, or a mixture of 25 µM OA with 50 µM or 200 µM PA, by 2-, 2.7- and 5.6-fold, respectively, compared with BSA-treated cells, while preserving hepatocyte health (Fig. 1B,C, green bars). Triacsin C, a selective inhibitor of long FA acyl-CoA synthetase, completely blocked lipid droplet accumulation in the presence of 150 µM OA, and decreased lipid droplet content by 50% in the presence of 1 µM TG, 25 µM OA and 200 µM PA cocktail (TG-FA) (Fig. 1B, red and green checkered bars, respectively).

Studies show that TG-induced ER stress leads to lipid accumulation in hepatic cells (Fang et al., 2013). To confirm whether the capacity to uptake FAs and/or to metabolize them is altered in response to induced ER stress in the hiPSC-Hep, a fluorescent FA analog labeled with BODIPY was tracked in the cells. Comparison between treatments of FA mix alone or FA in the presence of TG (TG-FA) showed that induced ER stress increased the exogenous FA analog accumulation, as measured by an increase in cell-associated fluorescence (Fig. 1D,E).

Considering that saturated FA-induced ER stress in hepatocytes has been reported to lead to cell death by apoptosis (Wei et al., 2006), cells were further characterized by measuring the activity of effector caspase 3/7 in treated hiPSC-Hep by live-cell imaging. No significant change in caspase 3/7 activity or nuclear area was evident in TG-FA-treated versus BSA-treated hiPSC-Hep after 18 h (Fig. 1F,G). Also using live-cell imaging, we tracked the accumulation of unfolded protein, a biochemical hallmark of ER stress, using 10 µM Thioflavin T, a small molecule that exhibits enhanced florescence when it binds to protein aggregates (Fig. 1H). Significant staining of aggregates was visible after 36 h of treatment with TG-FA, but not after 18 h. The time course for BODIPY immunofluorescence indicated that lipid accumulation in the presence of TG-FA increased over 36 h, but was not statistically significant beyond 12 h and did not affect valid cell count levels (Fig. S4A,B). In addition, the lipid spot signal-to-background ratio induced after 18 h was reproducible in two additional lots of hiPSC-Hep cells (Fig. S4C). These studies indicate that we can measure reproducible response to lipid accumulation and ER stress in hiPSC-Hep, without acutely inducing cell death by protein aggregation.

Having shown that inducing ER stress with TG contributes to increased lipid uptake into triglycerides in hiPSC-Hep, we wanted to investigate how repression of ER stress with tauroursodeoxycholic acid (TUDCA), an endogenous bile acid that is FDA approved to treat pediatric cholestatic liver disease, might prevent lipid uptake in our model. Several studies performed *in vivo* and *in vitro* indicate a crosstalk between inhibition of ER stress by TUDCA and reduction of triglyceride accumulation (Hamano et al., 2014; Beriault and Werstuck, 2013). Leptin-deficient (*ob/ob*) mice treated with TUDCA showed a decrease in liver fat content with Oil Red O histology and reduced expression of several genes involved in *de novo* lipogenesis (Yang et al., 2010). In cells, TUDCA suppressed ER stress-induced pPERK and XBP1 splicing (Ozcan et al., 2006), and blocked the lipid accumulation in PA-induced HepG2 cells by Oil Red O staining (Yan et al., 2018). We treated hiPSC-Hep with 500 µM TUDCA followed by TG-FA mixture for 18 h. Our results indicated that TUDCA treatment reduced TAG accumulation in the presence of 25 µM OA and 1 µM TG, and at a lower dose of PA (50 µM) plus OA and TG (Fig. S5A). As the concentration of PA is increased in the cocktail to 100 µM, the effect of TUDCA is slight and not statistically significant, and at 200 µM PA, the effect of TUDCA is masked completely. Cell viability was not affected in the nuclear channel (Fig. S5B). These results of an ER stressor and an ER inhibitor suggest a functional crosstalk between ER stress and intracellular lipid modulation.

### The UPR pathway is upregulated in the hiPSC-Hep model of steatosis

**Fig. 2.**
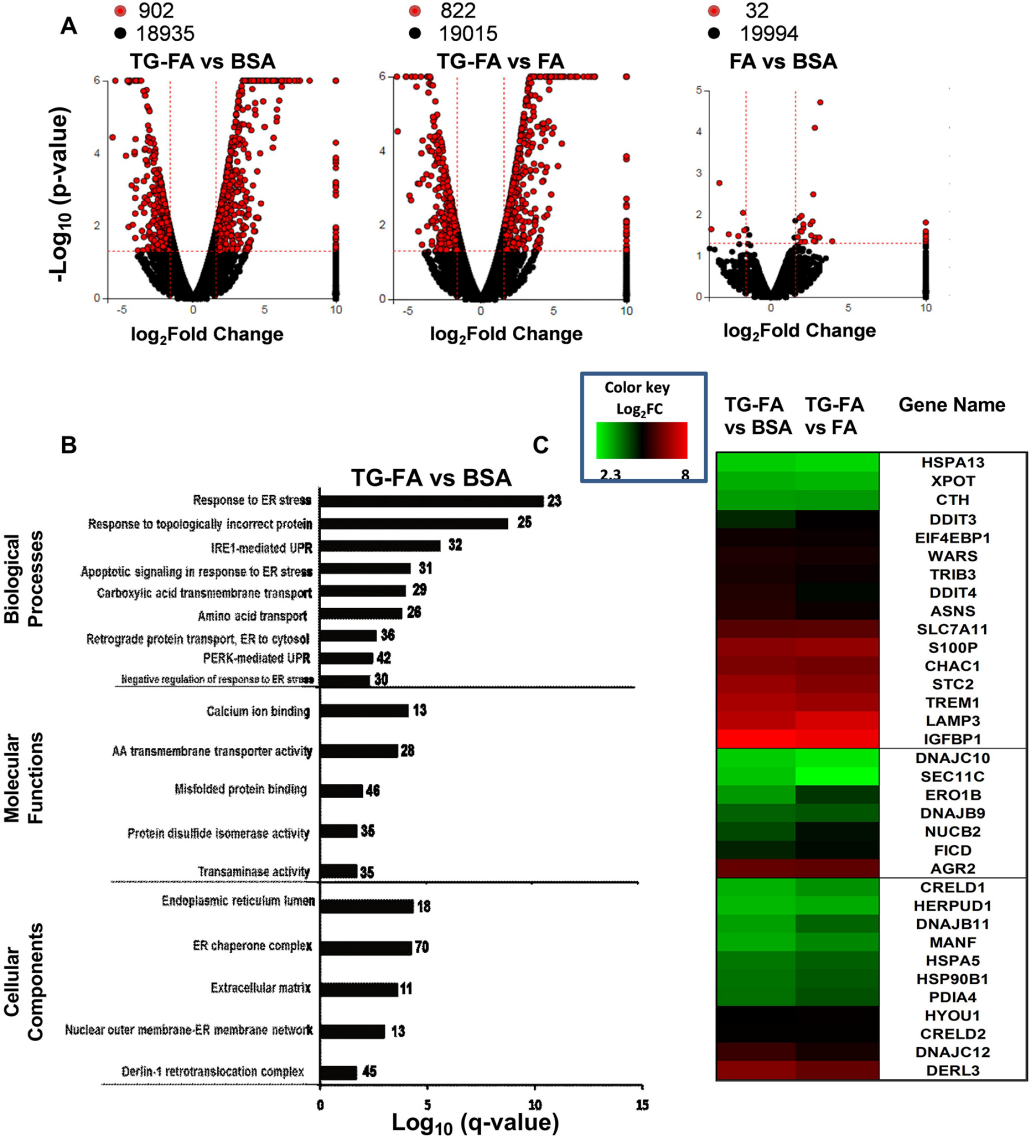
**Transcriptome and GO enrichment analysis.** (A) Global gene expression changes at the RNA level are shown in volcano plots of log_2_ fold change (log_2_FC) versus –log_10_ *P*-value for the three treatment groups. Red circles indicate global DEGs (*P*<0.05 with 1.6 log_2_FC cutoff); black circles denote non-DEGs. Data are representative of two independent RNA-seq determinations. (B) Bar chart representing the top over-represented GO categories with significant normalized enrichment scores according to the false discovery rate *P*-value (–log10 *q*-value cutoff 0.002), plotted relative to the pathway based on the measured expression changes induced across the pathway topology after treatment for 18 h with TG-FA, normalization to BSA and calculation by iPathwayGuide; the impact of each pathway is plotted relative to the number (noted to the right of bars) of DEGs enriched in each pathway. (C) Comparative heat map gene expression analysis of the effect of induced ER stress. DEGs associated with UPR pathways treated for 18 h with TG-FA were normalized to BSA (left column) or FA (right column) and color coded as log_2_FC from 2.3 to 8.0 with a *q*-value ≤0.05.

We identified genomic events associated with TG-FA treatment of hiPSC-Hep after 18 h by RNA sequencing (RNA-seq) analysis. Transcriptomic analysis was performed for control samples treated with BSA and test samples treated with 25 µM OA and 200 µM PA (FA) with or without 1 µM TG (TG-FA). Globally differentially expressed genes (DEGs) were identified as either up- or downregulated, with a *P*-value threshold of 0.05 between treatment groups, as shown by volcano plots (Fig. 2A). Treatment comparison groups revealed that induction of the gene set was a specific feature of TG-FA treatment. The most significantly enriched pathways filtered for false discovery rate (FDR) *q*-values ≤0.002 were associated with protein processing in ER, mineral absorption, metabolic pathways, drug metabolism and biosynthesis of amino acids (Table 1). The top Kyoto Encyclopedia of Genes and Genomes (KEGG) pathways linked to genes implicated in protein folding, ER-associated degradation and the pro-apoptotic marker CHOP (also known as DDIT3) are shown in Fig. S6.

**Table 1.**
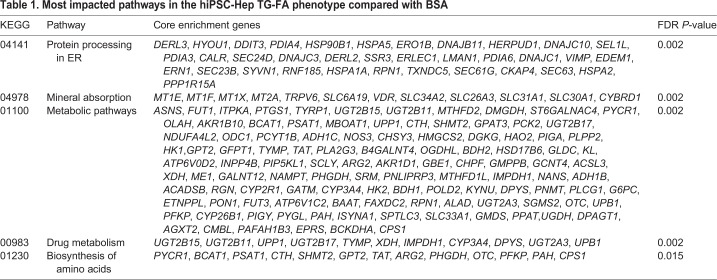
**Most impacted pathways in the hiPSC-Hep TG-FA phenotype compared with BSA**

In addition, enrichment analysis of the Gene Ontology (GO) by iPathwayGuide yielded terms pertinent to the activation of ER signaling. To obtain coherent functional modules, we chose terms on the percentage of gene products annotated to them and classified them with *q*-values ≤0.02 (Fig. 2B). Top overrepresented GO categories mapped to ER stress-mediated signaling pathways within the UPR and amino acid transport. Next, we ranked DEGs located downstream of the signaling branches of the UPR, including protein kinase R(PKR)-like endoplasmic reticulum kinase (*PERK*; also known as *EIF2AK3*), X-box binding protein 1 (*XBP1*) and activating transcription factor 6 (*ATF6*). Fold change values for genes upregulated in the TG-FA treatment group were normalized to the BSA and FA treatment groups for the gene set validated from a MEDLINE search (Table S2) and depicted in a heat map (Fig. 2C). The upregulation of the top-ranked genes suggested the activation of all three UPR arms which cooperate to maintain ER function.

A clinical study (Moylan et al., 2014) reported hepatic gene expression differences between 32 patients with severe NAFLD and 40 patients with mild NAFLD. Of the top 100 differentially expressed probes in the clinical study, 24 genes overlapped with our DEG data set and are listed in Table 2. Genes related to extracellular matrix organization and signal transduction (*COL1A2*, *COL14A1*, *EPHA3*) were upregulated in both studies. Markers of adult progenitor cells were upregulated in the clinical study but not in our results, with the exception of *SPP1*, a gene associated with NASH (Ryaboshapkina and Hammar, 2017). *EHF* is associated with fibrosis in NAFLD and is upregulated in both datasets; however, other genes associated with advanced fibrosis including *CXCL6*, *STMN2*, *UBD* and *DKK3* are upregulated in the clinical study but downregulated in our model, highlighting that our hiPSC-Hep cell system, which lacks the presence of other cell types such as stellate cells, is modeling the early steatosis stage but not advanced fibrosis, as expected. Downregulated genes in the clinical study were generally involved in metabolism and were not differentially expressed in our data set.

**Table 2.**
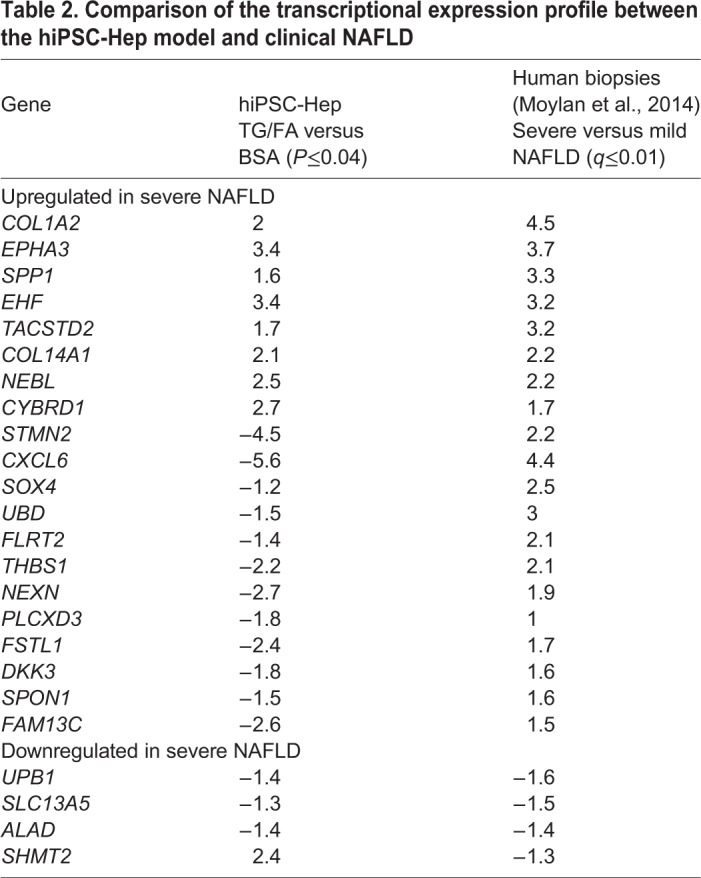
**Comparison of the transcriptional expression profile between the hiPSC-Hep model and clinical NAFLD**

**Fig. 3.**
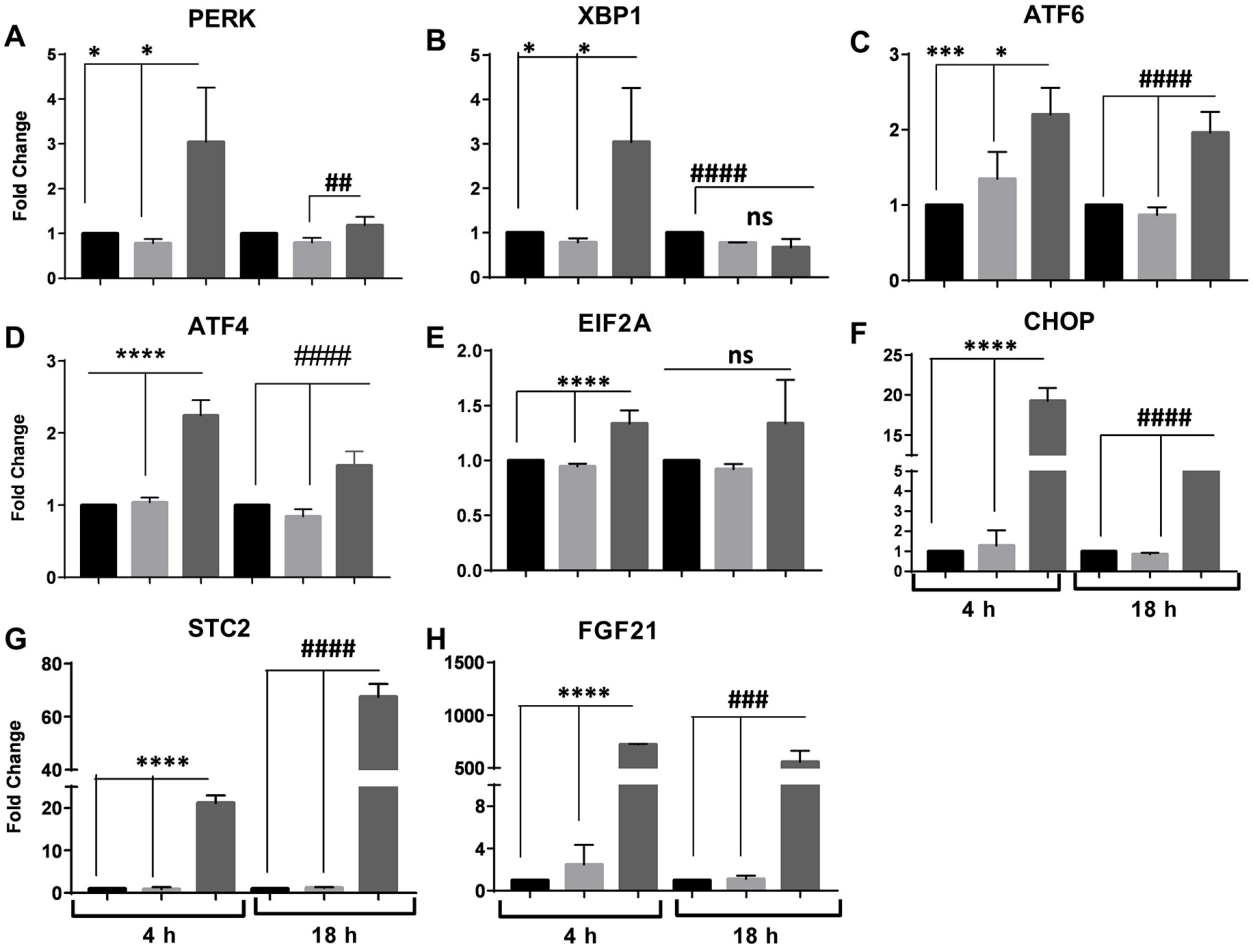
**Temporal expression of classic** **UPR genes.** (A-H) RT-qPCR analysis of genes involved in UPR response after 4 h and 18 h treatment with BSA (black bars), FA (light gray bars) or TG-FA (dark gray bars). Data are mean±s.d. of three experimental determinations, each performed with triplicate replicates. *P≤0.03; ***P≤0.005; ****P≤0.0001; ^##^*P*≤0.01; **^###^***P*≤0.005; **^####^***P*≤0.0001; ns, nonsignificant.

To confirm ER stress activation of the signaling branches in our model, we conducted RT-qPCR analysis of genes encoding transmembrane proteins and factors involved in UPR pro-survival pathways. Messenger RNA (mRNA) encoding the ER sensors PERK, ATF6 and XBP1 was moderately induced 2- to 3-fold after 4 h exposure to TG-FA. Considering the role of ATF6 in enabling cells to adapt to long-term ER stress, gene levels remained upregulated by 2-fold after 18 h treatment (Fig. 3A-C). Downstream of *PERK*, *ATF4* and *CHOP* (a gene involved in mediating apoptosis) were induced by 2- and 19-fold, respectively, after 4 h exposure to TG-FA, with no effect on *EIF2A*. At 18 h, *ATF4* and *CHOP* exhibited 1.5- and 5-fold attenuated expression over the control, respectively (Fig. 3D-F). Differences in expression levels in the presence of FA without TG were not significant, suggesting that the ER stress response is predominately activated by TG, as expected.

A central feature of our model is the cells’ adaptive response to ER stress, with the expression of genes that promote cell survival. As shown by RNA-seq and confirmed by RT-qPCR, stanniocalcin (STC2), a cytoprotective secreted protein (Ito et al., 2004), was upregulated 7-fold as a consequence of selective ATF4 activation; *STC2* was induced by 20- and 70-fold at 4 h and 18 h, respectively, indicating that PERK activation regulates both pro-survival and pro-apoptotic signaling during ER stress (Fig. 3G). Furthermore, we observed elevation of fibroblast growth factor 21 (*FGF21*) expression, a stress-responsive hepatokine that is activated during liver pathogenesis and injury and is associated with the occurrence of NAFLD in mice and patients (Li, 2011) (Fig. 3H). Expression of *FGF21* was upregulated 700- and 500-fold in TG-FA-treated hiPSC-Hep after early and late time points, respectively. *FGF21* expression is a response of functional hepatocytes to ER stress mediated by the IRE1 (also known as ERN1)-XBP1 branch of the UPR (Yang et al., 2013; Jiang et al., 2014). These data demonstrate protection of ER homeostasis by an adaptive mechanism of the three UPR pathways.

### Hepatic lipogenesis and metabolic dysfunction are dependent on induction of ER stress in hiPSC-Hep

**Fig. 4.**
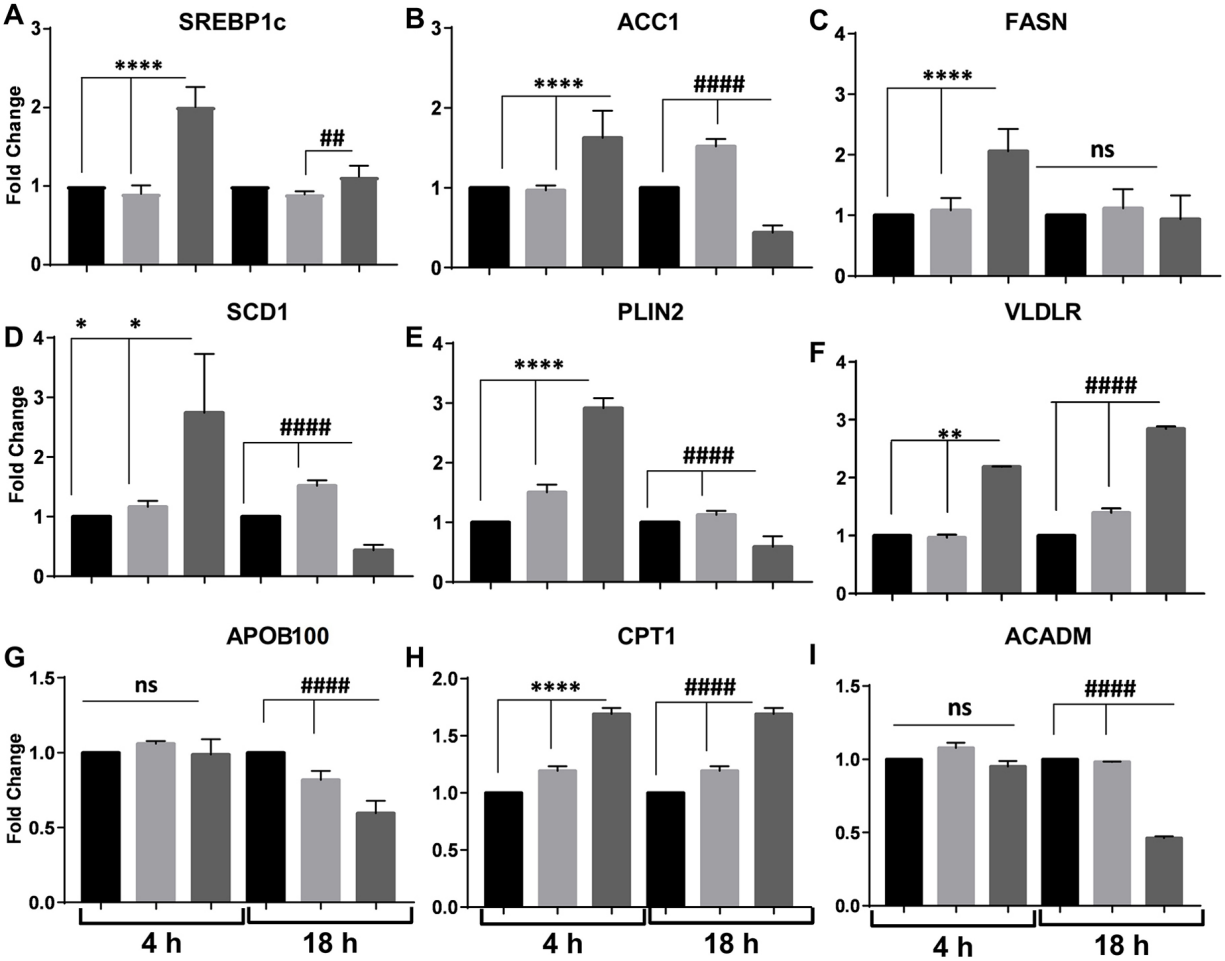
**Expression patterns of genes involved in lipid metabolism in response to ER stress.** (A-I) RT-qPCR analysis of genes involved in hepatic *de novo* lipogenesis, lipid export and mitochondrial β-oxidation after 4 h and 18 h treatment with BSA (black bars), FA (light gray bars) or TG-FA (dark gray bars). Data are mean±s.d of three experimental determinations, each performed with triplicate replicates. *P≤0.03; **P≤0.002; ****P≤0.0001; ^##^*P*≤0.01; ^####^*P*≤0.0001; ns, nonsignificant.

Bioinformatic cluster analysis of transcriptome data obtained from livers isolated from patients affected by NASH demonstrated an enrichment of downregulated genes in the ER stress-associated lipogenesis and UPR gene categories (Lake et al., 2014). We determined whether hepatic-induced ER stress was consistent with altered expression of key genes involved in *de novo* lipogenesis, lipid transport and oxidation in our hiPSC-Hep model. Studies have linked hepatic steatosis to ER stress-induced sterol regulatory element-binding protein 1c (SREBP-1c; also known as SREBF1) expression and activation (Ning, 2011; Kammoun, 2009), and we observed significant *SREBP-1c* upregulation after TG-FA early treatment (Fig. 4A). Downstream key genes that encode lipogenic enzymes, such as acetyl CoA carboxylase 1 (ACC1; also known as ACACA), FA synthase (FASN) and stearoyl-CoA desaturase (SCD1; also known as SCD), as well as perilipin 2 (PLIN2), a protein that promotes fat storage in lipid droplets, were upregulated by TG-FA challenge (Fig. 4B-E). These pro-lipogenic responses were mitigated after prolonged TG-FA challenge, indicating a shift to restore metabolic homeostasis. Our results support earlier studies showing that the PLIN2-peroxisome proliferator-activated receptor pathway, a marker for induction of steatosis, is upregulated in hepatocyte-like cells induced with OA (Graffmann et al., 2016), and that downregulation of PLIN2 is associated with ER stress resolution (Chen, 2017).

Analysis by RT-qPCR showed that mRNA levels for the VLDL receptor (VLDLR) were also significantly elevated at 4 h and continued to rise until 18 h (Fig. 4F). Consistent with our data, VLDLR expression is induced in hepatocytes *in vivo* under ER stress conditions and is responsible for intracellular triacylglyceride accumulation (Jo et al., 2013). The assembly and secretion of hepatic triacylglyceride-enriched VLDL that occurs in the secretory vessels of hepatocytes involves the transfer of triacylglyceride to apolipoprotein B100 (APOB100), a major protein component of VLDL. An additional phenomenon connecting aberrant lipid content and ER stress is decreased *APOB100* gene expression (Fig. 4G) and concomitant defective VLDL delivery as a result of impaired secretion at 18 h of APOB100 into the medium (data not shown) from hiPSC-Hep when challenged with TG-FA (Ota et al., 2008).

Finally, carnitine palmitoyltransferase 1 (*CPT1*; also known as *CPT1A*) mRNA, which encourages flux of FA through β-oxidation, was elevated in both treatment groups; but acyl-coenzyme A dehydrogenase (*ACADM*) expression, encoding an important enzyme for breakdown of medium-chain FA in mitochondria, was downregulated by 2-fold at 18 h, when cells were treated with TG-FA (Fig. 4H,I). Thus, in our hiPSC-Hep model, increased FA uptake, short-term upregulation of *de novo* lipogenesis, and impairment in FA oxidation or export might contribute to hepatic TAG accumulation.

### OCA reduces FA uptake and TAG synthesis in the hiPSC-Hep model of steatosis

**Fig. 5.**
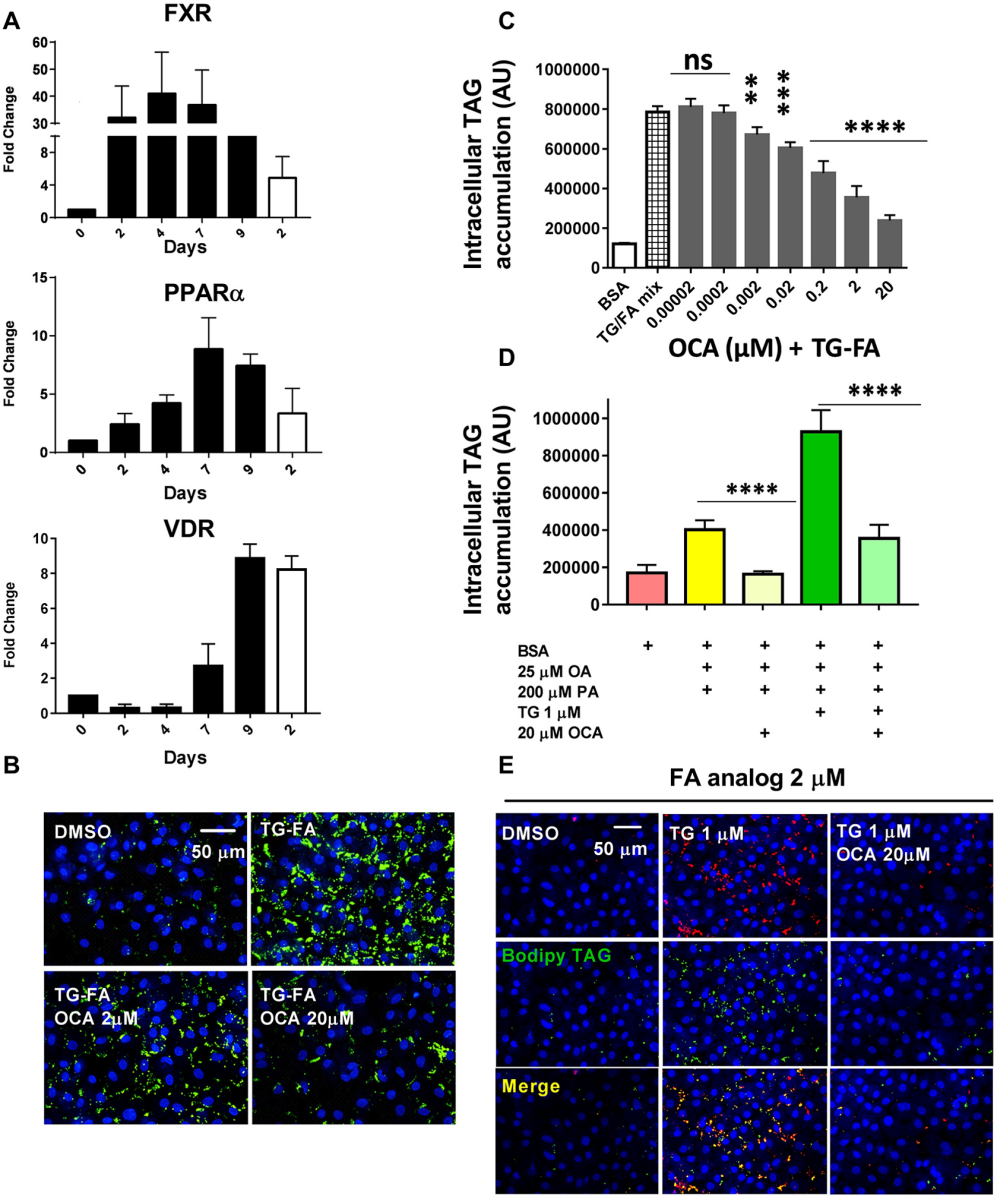
**OCA inhibits phenotype in the hiPSC-Hep model of steatosis.** (A) RT-qPCR analysis of key nuclear receptors expressed in hiPSC-Hep from day 0 to 9 in culture (black bars) and compared with primary hepatocytes at day 2 in culture (white bars). The fold induction values are relative to day 0. (B) BODIPY-labeled neutral lipids (green) show increased prevention of lipid accumulation by 2 µM and 20 µM OCA in TG-FA-treated cells compared with BSA-treated control cells. Nuclei are stained blue. Scale bar: 50 µm. (C) Titration of OCA in 384w showed dose-dependent reduction of lipid accumulation. Data are mean±s.d. of three experimental determinations, each performed with triplicate replicates. (D) An independent hiPSC-Hep line (donor 1279) showed similar lipid accumulation induced by FA (yellow bar) and TG-FA (dark green bar) as in Fig. 1A and Fig. S4A and C (donor 1434), and similar inhibition by 20 µM OCA as in C (light yellow and light green bars), compared with BSA-treated cells (pink bar). (E) OCA inhibits the uptake of exogenous fluorescent-labeled BODIPY-C_12_ FA analog and incorporation into TAG. BODIPY-C_12_ FA uptake (2 µM, red) by hiPSC-Hep and its incorporation into lipid droplets (green) occurred mainly in TG-treated cells (1 µM). As shown by merged staining (yellow, bottom row), BODIPY-C_12_ was incorporated into stained BODIPY 493/503-positive lipid droplets within 18 h (middle row), indicating that the FA analog was esterified for lipid droplet incorporation. 20 µM OCA co-treatment with BODIPY-C_12_ prior to treatment with 1 µM TG prevented FA uptake and TAG synthesis (right column). Scale bar: 50 µm. **P≤0.01; ***P≤0.002; ****P≤0.001; ns, nonsignificant.

The liver contains a network of nuclear receptor-regulated pathways that control lipid and glucose metabolism, bile acid homeostasis, inflammation and fibrosis (Rudraiah, 2016). During hiPSC-Hep *in vitro* maturation, we evaluated the expression of *FXR* (also known as *NR1H4*), peroxisome proliferator-activated receptor alpha (*PPARA*), and vitamin D receptor (*VDR*) compared with that of primary hepatocytes by RT-qPCR analyses (Fig. 5A). The results indicate that the nuclear receptors were highly expressed in hiPSC-Hep by day 7-9 compared with primary hepatocytes. FXR is agonistically controlled by bile acid to regulate a variety of target genes controlling lipid and sterol metabolism (Roda, 2017). We validated our model with the clinical compound OCA, a semisynthetic bile acid analog and selective FXR agonist (Neuschwander-Tetri et al., 2015). OCA dose dependently reduced hepatic TAG accumulation with a half-maximal inhibitory concentration (IC_50_) of 0.8±0.2 µM (*n*=3 when cells were pre-treated for 24 h and co-treated for an additional 18 h in presence of TG-FA) (Fig. 5B,C). OCA potency was not significantly different when cells were treated with FA mix in the absence of TG (IC_50_=0.6±0.2 µM, *n*=3; data not shown).

To validate our key results, we obtained commercially available hiPSC-Hep generated from a different individual than the donor line used to generate data in Figs 1-5 and Figs S1-S4. The cells were terminally differentiated and monitored by flow cytometry by a standard protocol, as described in the Materials and Methods. As shown in Fig. 5D, the cells produced a similar level of intracellular TAG accumulation, and produced a 2.4-fold and 5.5-fold increase in lipid for cells treated with FA mix alone or TG-FA, respectively, compared with BSA-treated cells (compare with Fig. 1B and Fig. S4A,C). Furthermore, 20 µM OCA treatment inhibited lipid accumulation induced by FA alone or by FA-TG by 100% and 75%, respectively (compare with Fig. 5C).

We chose to treat cells with OCA prior to treatment with TG-FA as a standard primary approach for drug screening to identify and evaluate compound prevention of lipid accumulation phenotype and subsequent transcriptomic changes. Our results are in accordance with therapeutic concentrations of OCA (3 nM to 3 µM) (Zhang et al., 2017) and with studies performed in a human liver *in vitro* system (0.5 µM) (Feaver et al., 2016). In addition, in a similar experiment to that shown shown in Fig. 1D, pre-treatment of cells with OCA inhibited the uptake of a tracer amount of exogenous fluorescently labeled FA analog (C1-BODIPY-C12) in the presence of TG and its subsequent incorporation into lipid droplets (Fig. 5E) (Rambold et al., 2015). Future drug characterization studies will test additional treatment modalities to differentiate a compound's ability to prevent lipid accumulation versus ability to clear steatosis following lipid accumulation.

**Fig. 6.**
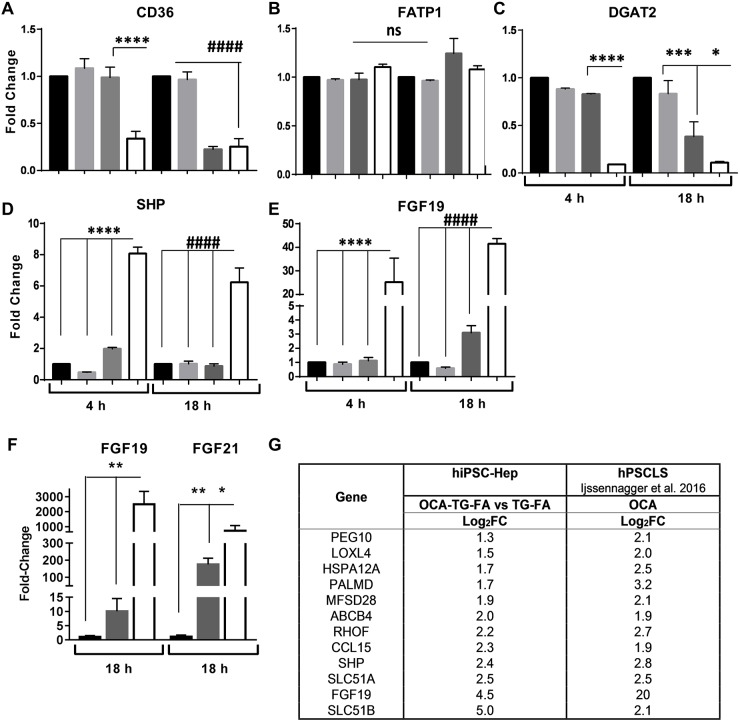
**FXR activation by OCA in hiPSC-Hep regulates the expression of target genes.** (A-E) RT-qPCR analysis of genes involved in TAG synthesis and FXR activation after 4 h and 18 h treatment with BSA (black bars), FA (light gray bars), TG-FA (dark gray bars) or 2 µM OCA (white bars). Data are mean±s.d. of three experimental determinations, each performed with triplicate replicates. (F) An independent hiPSC-Hep line (donor 1279) showed an increase in *FGF19* and *FGF21* gene expression induced by TG-FA (dark gray bars), and an increase in the presence of 20 µM OCA (white bars), similar to that shown in E and Fig. 3H for donor line 1434. (G) Comparative analysis of genes induced by OCA during TG-FA co-treatment. DEGs associated with FXR activation were normalized to TG-FA and reported as log_2_FC with *P*≤0.03. *P≤0.05; **P≤0.01; ***P≤0.005; ****P≤0.0001; **^####^***P*≤0.0001; ns, nonsignificant.

To study the role of OCA in the regulation of lipid accumulation, we pre-treated hiPSC-Hep with 2 μM OCA in the presence of TG-FA and assayed the expression of the FA translocase (CD36) that regulates the uptake of FA across the plasma membrane. *CD36* was downregulated at 4 h by greater than 2-fold and maintained up to 18 h (Fig. 6A), in accordance with a previous study in which activation of FXR by an agonist downregulated *CD36* expression in mice (Ma et al., 2013). Interestingly, *CD36* expression was repressed by TG-induced ER stress at 18 h. OCA treatment did not affect the expression of the gene encoding FA transport protein 1 (FATP1; also known as SCL27A1), indicating that CD36 expression is a specific response of OCA treatment (Fig. 6B). Downregulation of diacylglycerol acyltransferase 2 (DGAT2) by OCA by greater than 4-fold is evident at 4 h and maintained up to 18 h (Fig. 6C), in accordance with a previous study in an organotypic liver model (Dash et al., 2017).

In addition, we focused on OCA modulation of FXR target genes including small heterodimer partner (*SHP*; also known as *NR0B2*) (Fiorucci, 2004) and fibroblast growth factor 19 (FGF19) (Song et al., 2009). *SHP* and *FGF19* expression was upregulated by 2 µM OCA in the presence of TG-FA by 8- and 6-fold, and by 25- and 41-fold, after 4 h and 18 h, respectively (Fig. 6D,E), and was significantly higher than in the absence of OCA. The sensitivity of our assay to FXR agonist illustrates hiPSC-Hep competency for gene suppression and activation by the transcription factor FXR in regulating metabolic processes and functional relevance to drug testing for NAFLD progression. Again, we validated key findings in an independent donor hiPSC-Hep line (Fig. 6F). *FGF19* and *FGF21* expression was upregulated similarly in the presence of TG-FA compared with data in Figs 5E and 3H, respectively. Expression of both fibroblast growth factors was significantly upregulated in the presence of 20 µM OCA and TG-FA.

Finally, comparing our RNA-seq data with previously published data performed on human liver slices, we found similarity between gene signatures (Fig. 6G) (Ijssennagger, 2016). The most highly upregulated gene was *SLC51B*, which encodes the organic solute transporter-beta (OSTβ) and dimerizes with OSTα for bile acid disposal (Fig. S7). *FGF19* and *SHP* were also upregulated in both data sets, validating our results.

## DISCUSSION

Obtaining functional hepatocytes from hiPSCs that more closely approximate the functional characteristics of human primary liver samples and can be induced to manifest the pathobiology of liver disease, but are available at high purity and scale for drug discovery and development, is a major challenge (Zeilinger et al., 2016). Differentiation of hiPSCs into functional hepatocytes that exhibited classic hepatocyte-associated biofunctions, such as glycogen storage, albumin and urea secretion, and metabolic activities of cytochrome P450, consistent with those identified in primary hepatocytes, has been reported (Lu et al., 2015; Sirenko et al., 2014). In this study, we developed an *in vitro* drug discovery platform using hiPSC-Hep, which was induced to manifest features of pathobiology of liver steatosis. We showed that hiPSC-derived hepatocytes, as a mono cell type, displayed mature hepatocyte markers, abundant nuclear receptor expression and lipid metabolic activity, and provided a monolayer of cells compatible for high-content imaging to model molecular mechanisms associated with an increased risk of NAFLD. We validated key results with an independent hiPSC-Hep line with a different genetic background, showing similar lipid accumulation and gene expression levels for the NAFLD biomarker, *FGF21*, in the presence of FA and TG cocktail. Furthermore, we validated our model with OCA treatment, a clinical stage therapeutic for NASH, and transcriptomic data corroborated with reported clinical observations. OCA activity was also similar in the independent hiPSC-Hep donor line.

A pathogenic model for the progression from NAFLD steatosis to NASH is presented as a ‘two-hit’ hypothesis, whereby accumulation of TAG induces vulnerability of hepatocytes to factors such as ER stress and abnormal lipid metabolism, and promotes inflammation and triggers fibrogenesis (Day, 1998; Walsh et al., 2004). In our study, hiPSC-Hep subjected to lipid overload and challenged with acute induced-ER stress developed a steatotic phenotype, as shown by the number of DEGs after FA treatment in combination with TG challenge. Our results confirmed that induced ER stress in the presence of an FA cocktail potentiated lipid accumulation through dysregulation of molecular mediators of lipid metabolism. Furthermore, the pharmaceutical chaperone TUDCA attenuated ER stress-induced lipid accumulation in hiPSC-Hep in the presence of TG, OA and low-dose PA, suggesting a crosstalk between ER stress and *de novo* lipogenesis. Finally, molecular phenotyping revealed upregulation of the UPR regulatory network and downstream targets, consistent with activation of compensatory mechanisms to restore homeostasis and promote cell survival and adaptation (Henkel and Green, 2013).

Previous studies suggested that PA at concentrations relevant to *in vivo* conditions reduced Ca^2^^+^ stores in the ER, but co-incubation with unsaturated FA reduced the induction of ER stress and cell death (Listenberger et al., 2003; Wei et al., 2006, 2009). Exposure of hiPSC-Hep to a PA and OA mixture and TG caused a nearly 6-fold increase in TAG accumulation, revealing a comparable increase in hepatocytes isolated from steatotic human livers versus those obtained from healthy livers (Amaro, 2010). Intercellular lipid accumulation was associated with significant upregulation of the expression of genes in the ER stress-UPR axis, indicating activation of response signaling pathways responsible for alteration in lipid homeostasis that underlies the steatosis *in vitro*. The transcriptomic analysis revealed 34 modified genes related to the three ER stress branches: *PERK*, *IRE1/XBP1* and *ATF6*. RNA-seq analysis suggests that the modulation of the ER stress-UPR network was biologically significant, as shown by functional enrichment with the KEGG pathway database and GO database.

Validation by RT-qPCR revealed time-dependent expression dynamics of DEGs upon TG-FA challenge. The expression of key ER stress markers was inducible at 4 h and reduced at 18 h post-treatment, indicating an adaptive response to the exogenous insults and transcriptional reprogramming to restore homeostasis and protect cells from apoptosis. Notably, we observe increased expression of *STC2*, a pro-survival component of UPR (Ito et al., 2004), and *FGF21*, a marker of hepatic fat and protector of NAFLD-induced adverse effects such as ER stress (Jiang et al., 2014); both targets are increased in human NASH (Lake et al., 2014; Li, 2010).

An additional goal of our phenotypic model was to induce dysregulation of genes involved in *de novo* lipogenesis, TAG synthesis and lipid droplet formation known to be involved in the development and progression of NAFLD (Berlanga et al., 2014; Koo, 2013). Top GO pathways upregulated in OA-induced hepatocyte-like cells belonged to lipid metabolism and transport (Graffmann et al., 2016). The majority of genes upregulated in our model at 4 h and reduced at 18 h post-treatment indicated that an ER stress-associated negative feedback regulation exists for genes involved in lipid metabolism. A recent study highlighted the contribution of genomic reprogramming by the UPR to reduce the expression of *de novo* lipogenesis-related genes to alleviate continued lipotoxic stress and pathological damage during NASH (Lake et al., 2014). Activation of the PERK-ATF4 pathway under ER stress condition is required for hepatic VLDLR upregulation in hepatocytes, which is responsible for hepatic steatosis (Jo et al., 2013). Upregulation of *VLDLR* and downregulation of *APOB100* gene expression at 18 h in our model might underlie elevated lipoprotein delivery and diminished export of lipids, respectively, to and from hiPSC-Hep during ER stress. Furthermore, mitochondrial β-oxidation is the primary oxidative pathway for the disposal of FA through esterification to CPT I, II. Acyl-CoA is then catalyzed to ACADM. RT-qPCR analysis showed increased *CPT1* gene expression and reduced *ACADM* levels at 18 h, indicating that FA oxidation repression potentially worsens the steatotic phenotype.

We challenged steatosis stimuli-induced hiPSC-Hep with OCA, a FXR agonist with antisteatotic and inflammatory properties (Mi, 2003; Cipriani, 2010; Ali, 2015). OCA showed benefit to NAFLD/NASH patients in clinical trials, as well as improvement in phenotype and gene signature in liver-based *in vitro* models (Neuschwander-Tetri et al., 2015; Feaver et al., 2016; Maneschi et al., 2013). In our results, OCA dose dependently reduced TAG accumulation with an IC_50_ of 0.8 µM, similar to published data (Feaver et al., 2016). Expression of the transport gene *CD36*, the protein activity of which is crucial for development of steatosis in obese patients with liver disease (Miquilena-Colina et al., 2011), was significantly reduced by OCA. Also, improvement of hepatic steatosis by *CD36* downregulation in a murine model of NAFLD was reported for another FXR agonist, GW4064 (Ma et al., 2013), which is also an inhibitor in our phenotypic model, with similar potency to OCA, albeit lower efficacy (data not shown). Expression of *DGAT2*, which encodes an enzyme that catalyzes the final step in triacylglyceride synthesis was also significantly reduced by OCA, and studies indicate that knockdown of *DGAT2* over *DGAT1* significantly reduces hepatic lipids (Choi et al., 2007). These changes were also associated with reduced expression of the lipogenic genes *SREBP-1c*, *ACC1* and *SCD1*, which are upregulated at 4 h by TG-FA treatment in our model.

Although data suggest that OCA has a selectivity to another described bile acid receptor, G protein-coupled bile acid receptor 1 (Bowlus, 2016), the receptor seems not to be expressed in hepatocytes (Keitel and Häussinger, 2012). In fact, it has been proposed that the bile acid receptor could indirectly affect liver function and triglyceride metabolism through the involvement of other cell types of the liver not included in our model. Then, our results indicate that the beneficial effect of OCA is specifically mediated by FXR pathway activation in hiPSC-Hep treated with TG-FA. Indeed, we found that the expression of the FXR target genes *SHP* and *FGF19* is upregulated by OCA treatment (Watanabe et al., 2004; Miyata et al., 2011). We also show that *FGF19* is upregulated in a dose-dependent manner by OCA in an independent donor hiPSC-Hep line.

Although there is an emergence of therapeutic development, there are currently no FDA-approved medicines to treat NAFLD and the varying pathogenic mechanisms that manifest in the patient to result in NASH (Filozof et al., 2015; Vinod et al., 2016; Gawrieh and Chalasani, 2015). By recapitulating *in vitro* characteristic features of progressive steatotic liver disease in hiPSC-Hep and demonstrating translatability of the cell phenotype with disease mechanism, we propose our model as a useful drug discovery platform to identify and evaluate potential new therapeutics. To the best of our knowledge, this is the first study demonstrating the experimental pathology in hiPSC-Hep for NAFLD that can be used for high-throughput drug screening and pharmacology in conjunction with additional *in vitro* complex systems (Feaver et al., 2016).

An added advantage to using hiPSC technology is the potential to incorporate genotype-specific cells derived from individual patient samples into the drug discovery process. For example, patients with the patatin-like phospholipase domain-containing 3 gene (*PNPLA3*), a single nucleotide polymorphism in a TAG lipase, are associated with a high risk for NASH. Studies suggest a potential role of the mutant protein in hepatic fat metabolism and TAG accumulation through the regulation of XBP1 expression under ER stress (Ochi et al., 2016). hiPSC lines derived from these patient tissue samples, a current goal of Cellular Dynamics International (CDI), can be compared with our comprehensive data set and incorporated into the drug discovery process, to aid in the pharmacogenomics of drugs of potential use in the treatment of NAFLD/NASH and to better understand molecular mechanisms related to genetic background in NAFLD progression.

## MATERIALS AND METHODS

### hiPSC-Hep cell culture and compound treatment

Authenticated cryopreserved hiPSC-Hep from individual female donors were purchased commercially (1434 and 1279, CDI, Madison, WI, USA). All experiments were conducted in iCell Hepatocytes 2.0 (hereafter referred to as hepatocytes 2.0) and, unless otherwise noted, experiments were conducted in donor line 1434. According to the manufacturer, the pluripotent stem cells are differentiated to induced pluripotent stem cell-derived hepatocytes 2.0 by directed differentiation, employing sequential addition of small molecules and growth factors. The proprietary process is monitored by marker expression characterization at the stages of endoderm formation, through hepatoblast specification and terminal differentiation to hepatocytes as determined by flow cytometry of alpha-1 antitrypsin expression (Lu et al., 2015). Upon receipt, the hepatocytes 2.0 were thawed, plated at a density of 2.2×10^4^cells/well (cell line 1434) or 3.6×10^4^cells/well (cell line 1279) on collagen I-coated 384w plates (BD Biosciences, San Jose, CA, USA); and allowed to mature post-thaw over 7-9 days of culture, as monitored by increasing albumin expression, according to vendor specifications, with minor modifications. In brief, cells were cultured in RPMI 1640 medium plus glutamine (Thermo Fisher Scientific, Waltham, MA, USA) containing 1× B27 (Thermo Fisher Scientific), 20 ng/ml oncostatin M (R&D Systems, Minneapolis, MN, USA), 0.1 µM dexamethasone (Sigma-Aldrich, St. Louis, MO, USA), 25 µg/ml gentamicin (Thermo Fisher Scientific) and hepatocytes 2.0 medium supplement (CDI). Oncostatin M, a member of the interleukin-6 cytokine family, in combination with dexamethasone, is required for differentiation (Hannan et al., 2013; Carpentier et al., 2016). After 5 days of culturing, hepatic maturation was maintained with medium William's E medium (Thermo Fisher Scientific) supplemented with 0.1 µM dexamethasone and hepatocyte maintenance supplement pack (Thermo Fisher Scientific), containing insulin-transferrin-selenium. These compounds are used to maintain the *in vitro* function of hepatocytes and promote differentiation to the hepatic lineage (Kamiya et al., 1999; Tomizawa et al., 2013). Medium was replaced every 24 h with a 384-channel electronic pipette (Integra, Viaflo, Hudson, NY, USA).

For lipid induction in hiPSC-Hep, cells were treated with 15 µl of a 2× mixture of OA (Sigma-Aldrich), and PA (Agilent Technologies, Santa Clara, CA, USA) or BSA in 30 µl. FAs were used at 8:1 ratio of PA to OA. TG (Abcam, Branford, CT, USA) was added in the presence and absence of FA and incubated for 18 h at 1 µM. Triacsin C (Enzo Life Sciences, Farmingdale, NY, USA) was added at a 5 µM final concentration in the presence of FA. TUDCA (580549, MilliporeSigma, Burlington, MA, USA) was added at a 500 µM final concentration. Cells were washed with PBS and incubated for 15 min with BODIPY 493/503 (8 µg/ml, Thermo Fisher Scientific) at 37°C to label lipid droplets. Cells were washed with PBS and fixed with 4% paraformaldehyde at room temperature (RT). Nuclei were labeled with 10 µg/ml Hoechst 33342 (Thermo Fisher Scientific) and plates were imaged and analyzed as described below in the ‘High-content imaging and quantification’ section. HiPSC-Hep conditioned medium was collected and stored at −20°C until assayed. Albumin and urea production was quantified after 24 h and 48 h in fresh culture medium using human ELISA quantification kits (Abcam), according to the manufacturer's protocol, and read on an EnVision microplate reader (Perkin Elmer, Waltham, MA, USA). The concentrations of albumin and urea were normalized to the number of total cells determined from each well.

For dose-response titration, OCA (Abcam) stocks at 20 mM and 0.312 mM in 100% dimethyl sulfoxide (DMSO) were used. From each stock concentration, 2-fold serial dilutions were performed in 2.5 nl increments by acoustic dispensing using Echo 555 (Labcyte, Sunnyvale, CA, USA) and pre-incubated with cells for 24 h. The final concentration of DMSO did not exceed 0.25% in all wells. Data were fit to nonlinear regression with variable slope to determine half-maximal effective concentration (EC_50_) values, or to one-way analysis of variance (ANOVA) to determine column statistics between treatment groups using Prism 7.0 software (GraphPad, San Diego, CA, USA).

### Primary human hepatocyte cell culture

Cryopreserved ‘5-Donor’ plateable human hepatocytes (Thermo Fisher Scientific), according to the manufacturer's instructions, were transferred into 50 ml cryopreserved hepatocyte recovery medium (Thermo Fisher Scientific) and centrifuged at 100 ***g*** for 10 min at RT. Then, the cell pellet was resuspended by gently pipetting up and down in plating medium, William’s E medium supplemented with hepatocyte plating supplement pack (Thermo Fisher Scientific), 1 µM dexamethasone (Thermo Fisher Scientific) and 5% fetal bovine serum. Cells were plated onto collagen I-coated plates to achieve full confluency. After 5 h, nonattached cells were washed away and the plating medium was replaced with William’s E medium supplemented with hepatocyte maintenance supplement pack and 0.1 µM dexamethasone. After 24 h and 48 h, mRNA was extracted and gene expression analysis was carried out by RT-qPCR.

### High-content imaging and quantification

All images were acquired on an Operetta Imaging System (Perkin Elmer) with each channel acquired sequentially: Hoechst 3342 (nuclei) (Thermo Fisher Scientific) using 365 nm Xenon lamp excitation and 450/50 nm emission filters; Alexa Fluor 488 using 488 nm laser excitation and 540/70 nm emission filters. Images were acquired using Harmony software and transferred to a remote server (Columbus Image Data Management System, Perkin Elmer) and analyzed using algorithms developed in the Acapella 2.6/2.7 High-Content Image Analysis software package (Perkin Elmer).

Quantification of lipid induction was performed as follows. First, the nuclei were detected from the Hoechst channel images using an Acapella standard nuclei detection module. From the detected nuclei, whole-cell borders were defined using an Acapella standard cytoplasm detection module. Within the detected cytoplasm region, TAG droplets were detected in the BODIPY channel using an intensity threshold. From the nuclei, cytoplasm and TAG images, multiple parameters were calculated for each cell. These ‘per cell’ results were statistically aggregated to yield cell population means and medians and standard deviations for each well. The calculated parameters of the assay included cell count per well and well averages of the ‘per cell’ values of nucleus area, roundness and average Hoechst intensities, as well as integrated TAG droplet area and average BODIPY green intensities of the TAG regions in the cytoplasm/cell. The well average of the integrated spot signal BODIPY green intensity of the TAG region in the cytoplasm was selected as the primary assay readout because it correlated with the overall uptake of TAG per cell. To quantify uptake of exogenous lipid, 2 µM BODIPY 558/568 C_12_ (Life Technologies, Carlsbad, CA, USA) was incubated overnight with cells in William's E medium in the absence or presence of 20 µM OCA, followed by addition of TG for 24 h. Cells were labeled with BODIPY 493/505 and Hoechst and imaged as described.

### Live-cell imaging

Activation of caspase activity in treated cells was measured using CellEvent Green Detection Reagent (Life Technologies), a fluorogenic substrate for activated caspase 3 and 7 (Antczak, 2009). hiPSC-Hep were treated with TG-FA mixture and incubated with 5 μM substrate for 30 min at RT, followed by addition of 1 µg/ml Hoechst 3342. Live-cell images (three fields per well in triplicate wells) were captured with an Operetta Imaging System using standard filter sets and analyzed using Columbus software. Cell counts per well and well averages of ‘per cell’ values of nucleus area, roundness and average Hoechst intensities, as well as integrated intensities in the Caspase Green channel, were calculated. Staurosporine at concentrations from 0.125 to 1 μM was used as a positive control for caspase activation after 18 h incubation with cells. Accumulation of unfolded protein aggregates was measured using Thioflavin T staining. hiPSC-Hep were co-incubated with TG-FA mixture and 10 μM Thioflavin T dye (Acros Organics, Morris Plains, NJ, USA) followed by addition of 1 µg/ml Hoechst 3342. Live-cell images were captured with an Operetta Imaging System using standard filter sets.

### RNA-seq and RT-qPCR

RNA was isolated from primary hepatocytes and hiPSC-Hep using an RNeasy kit (Qiagen, Frederick, MD, USA) and subsequently subjected to DNase Digestion (Qiagen). Total RNA was quantified using NanoDrop 8000 (Thermo Fisher Scientific). The quality of total RNA was assessed by an Agilent Bioanalyzer Nano Chip (Agilent Technologies) and the RNA integrity number (RIN) ranged from 9.7 to 10. Total RNA (1 µg) was used as starting material to construct an RNA-seq library using a Truseq Stranded Total RNA Library preparation kit (Illumina, San Diego, CA, USA). First, ribosomal RNA (rRNA) was removed from total RNA and the remaining non-rRNA was fragmented into small pieces using divalent cations under elevated temperature. Following fragmentation, the first complementary DNA strand (cDNA) was synthesized using random primers, followed by second-strand synthesis using DNA polymerase I. The cDNA was then ligated with index adapters for each sample followed by purification, and enriched with PCR to create the final library. The quality and quantity of the libraries were detected by Agilent Bioanalyzer and Kapa Biosystems qPCR. Multiplexed libraries were pooled and single-end 50 bp sequencing was performed on one flow cell of an Illumina Hiseq 1500.

RNA-seq raw data (reads) were quality controlled by FastQC. Mapping to human genome (hg38) was performed by tophat2 (Kim et al., 2013). SAMTools (Li et al., 2009) was used to select only mapped reads. Alignment files (.bam) were than imported into Partek NGS (Partek, St Louis, MO, USA) tool to provide data quantification, including computing read counts per gene and transcript and conversion to reads per kilobase per million (RPKM) data. The feature summarization step in Partek uses an expectation-maximization (EM) approach to estimate transcript abundance. The RPKM signals extracted are further log_2_ transformed to create data sets with signal distributions closer to a normal distribution. Box and whisker plots are used to verify the absence of outliers in the sample distributions. Additional quality control and clustering of the dataset was performed with principal component analysis (PCA). Read counts per gene in all samples were saved in a text file. DEGs between biological groups of interest were defined using DEseq procedure in R (Anders and Huber, 2010). In brief, DEseq normalizes raw read counts per gene and accounts for differential expression based on the negative binomial distribution model.

Reverse transcription was performed using a high-capacity cDNA reverse transcription kit (Applied Biosystems, Foster City, CA, USA), according to the manufacturer's recommendations, and the cDNA obtained was used for real-time quantitative PCR. Reaction mixture (10 μl) containing 1 μl cDNA template, 0.5 μl each of a primer and probe mix (20×, Thermo Fisher Scientific) for the gene of interest, the reference gene and TaqMan Universal PCR master mix (Applied Biosystems) were added to the opaque white 384-well plates and amplified as follows: 2 min of initial incubation at 50°C and 10 min of initial denaturation at 95°C, 45 cycles at 95°C for 15 s, 60°C for 1 min for annealing and 1 s at 72°C for final extension. Assays were designed to have primers/probes to span exon-exon junctions (Table S1). Direct detection of PCR products was monitored by measuring the fluorescence produced by the result of TaqMan probe hydrolysis after every cycle. All experiments were carried out in triplicate in a LightCycler 480 thermocycler (Roche Diagnostic). Relative gene expression from qPCR data between groups of samples was assessed using a ΔΔCq method (Livak and Schmittgen, 2001). To adjust for variations in the cDNA synthesis each gene was normalized to hypoxanthine phosphoribosyl transferase 1 (*HPRT1*) (*ELF2A*, *FGF21*, *ACC1*, *PLIN2*, *ACADM*, *CD36*, *FATP1*, *FGF19*, *VDR*, *PGC1A*, *PPARA*, *PPARG*, *CYP1A2*, *CYP2C8*, *CYP2B6*, *SOX17*, *POU5*, *TAT*, *HNMT*, *ABCB11*, *SLC22A* and *KRT8*) or to *GAPDH* mRNA (*PERK*, *ELF2AK3*, *XBP1*, *ATF6*, *ATF4*, *STC2*, *CHOP*, *SREBP-1c*, *FASN*, *SCD1*, *VLDLR*, *APOB*, *CPT1*, *DGAT2*, *NR0B2*, *FXR*, *PPARA*, *LXR*, *CYP3A4*, *CYP2C9*, *CYP2C19*, *AFP*, *TDO2*, *HNF4*, *ALB* and *CHD1*).

### Data and statistical analyses

High-content assay performance was evaluated in terms of signal-to-background ratio and statistical significance was calculated using two-tailed Student's *t*-test. RNA-seq data were analyzed by iPathwayGuide (Advaita Bioinformatics: http://www.advaitabio.com/ipathwayguide.html). Transcriptome data were uploaded to the NCBI Gene Expression Omnibus (GEO) (accession number GSE116185). Volcano plots rely on double-filtering criterion and display unstandardized signal (log_2_ fold change) against noise-adjusted/standardized signal (*P*-value). DEGs were normalized to the control group and reported as log_2_ of the fold change (log_2_FC). Unless otherwise indicated, fold induction values were averaged for all experiments performed as experimental triplicates and data are presented as mean±s.d. relative to BSA for each incubation time. Column statistics between control and treatment groups were analyzed by unpaired Student's *t*-test. Fold-change expression values obtained from RT-qPCR were analyzed by one-way analysis of variance (ANOVA) followed by post hoc comparisons of group means with the Tukey's multiple comparison tests at an overall confidence level of 95% using Prism software (GraphPad).

## Supplementary Material

Supplementary information
