# Supplementary material for: A nonalcoholic fatty liver disease model in human induced pluripotent stem cell-derived hepatocytes, created by endoplasmic reticulum stress-induced steatosis
Source: Dis Model Mech. 2018 Sep 25;11(9):dmm033530. doi: 10.1242/dmm.033530 (PMC6176998; doi:10.1242/dmm.033530)
Supplement: Supplementary information [file dmm-11-033530-s1.pdf]

Table S1. Genes used in the RT-qPCR analysis and their corresponding TaqMan gene expression assay IDs

Gene	Assay ID	Exon Boundary Range	Amplicon Length
ATF6	Hs00232586_m1	6-7	95
ATF4	Hs00909569_g1	1-4	68
PERK	Hs00984006_m1	3-4	83
XBP1s	Hs00231936_m1	3-4	60
CHOP	Hs00358796_g1	2-4	93
STC2	Hs01063215_m1	3-4	93
EIF2α	Hs00230684_m1	8-11	106
SRBP1c	Hs01088691_m1	6-7	90
ACC1	Hs01046047_m1	29-33	65
SCD1	Hs01682761_m1	2-3	129
FASN	Hs01005622_m1	3-4	62
PLIN2	Hs00605340_m1	5-7	139
ApoB	Hs00181142_m1	20-21	69
FAT-CD36	Hs00354519_m1	3-5	83
FATP1	Hs01587911_m1	11-12	120
FGF19	Hs00192780_m1	2-3	54
FGF21	Hs00173927_m1	3-4	117
CPT1α	Hs00912671_m1	11-12	75
ACADM	Hs00936584_m1	5-10	98
SHP	Hs00222677_m1	1-2	87
VLDLR	Hs01045922_m1	16-19	98
FXR	Hs01026590_m1	11-12	78
PPARα	Hs00947536_m1	4-6	62
PPARβ	Hs04187066_g1	5-8	69
PPARγ	Hs01115513_m1	4-6	90
LXR	Hs00172885_m1	6-8	78
VDR	Hs00172113_m1	2-4	62

Disease Models & Mechanisms • Supplementary information

Table S2. Gene set validated from a MEDLINE search

ER Branch	Gene	TG/FA vs BSA		Description	References
		Fold change	q-value		
PERK	*IGFBP1*	8	6.5E-17	Insulin-like growth factor (IGF)-binding protein-1	J Biol Chem.2006 14;281(28):19124-33.
	LAMP3	6.7	5.8E-12	Lysosomal associated membrane protein 3	Breast Cancer Res. 2013; 15(1):R2
	TREM1	6.4	1.4E-10	Triggering receptor expressed on myeloid cells 1	Brain Behav Immun. 2011; 25(6):1162-9
	STC2	6.1	4.4E-11	Calcium- and phosphate-regulating hormone	Mol Cell Biol. 2004; 24(21): 9456–9469.
	CHAC1	5.6	3.7E-9	Cation-transport regulator homolog 1	J Biol Chem. 2015; 290(25):15878-91.
	S100P	5.8	4.6E-10	S100 Calcium Binding Protein P	J Biol Chem. 2009;284(7):4158-67.
	SLC7A11	4.8	7.5E-8	Solute carrier family 7/member 11 (Cystine/glutamate antiporter)	J Biol Chem. 2009;284(2):1106-15
	ASNS	3.9	1.7E-5	Asparagine synthetase	Mol Biol Cell. 2016; 27(9): 1536–1551.
	DDIT4	3.8	2.9E-5	DNA-damage-inducible transcript 4/REDD1	Mol Biol Cell. 2016; 27(9):1536-51; Biochem Biophys Res Commun. 2012; 427(3): 485–489
	TRIB3	3.6	8.7E-5	Tribbles Pseudokinase 3	EMBO J. 2005; 24(6): 1243–1255.
	WARS	3.7	4.2E-5	Aminoacyl tRNA-synthetases	Nat Cell Biol. 2013; 15(5): 481–490.
	EIF4EBP1	3.4	0.0002	Eukaryotic translation initiation factor 4E-binding protein 1	Cell Metab. 2008; 7(3):269-76
	CHOP	3	0.002	C/EBP homologous protein (CHOP)/growth arrest and DNA damage-inducible gene 153 (GADD153)/DDIT3	J Lipid Res. 2016; 57(8):1329-38.
	CTH	2.5	0.02	Cystathionine γ-lyase	Mol Biol Cell. 2016; 27(9): 1536–1551.
	XPOT	2.4	0.02	Exportin for tRNA	
	HSPA13	2.3	0.05	heat shock protein family A (Hsp70) member 13/STCH	Physiol Genomics. 2009; 38(3):328-41
XBP1s	*AGR2*	5.1	4.3E-8	Anterior gradient 2, protein disulphide isomerase family member	J Biol Chem. 2011; 286(52): 44855–44868
	FICD	3	0.002	FIC domain containing/Hype	J Biol Chem 2015; 290(13): 8482–8499
	NUCB2	2.9	0.004	Nucleobindin 2	FEBS Letters. 2006; 580(1):184–190
	DNAJB9	2.7	0.007	DnaJ heat shock protein family (Hsp40) member B9/ERdj4	Cell Rep. 2013;3(4):1279-92
	ERO1B	2.5	0.02	Endoplasmic reticulum oxidoreductase 1 beta	Cell Rep. 2013;3(4):1279-92
	SEC11C	2.3	0.04	SEC11 homolog C, signal peptidase complex subunit	Cell Metab. 2012;16(4):487-99.
	DNAJC10	2.3	0.05	DnaJ heat shock protein family (Hsp40) member C10/ERdj5	Nat immunol. 2014; 15(3):248-57
ATF6	*DERL3*	5.7	6.4E-9	Derlin 3	Dev Cell. 2008 Dec;15(6):829-40
	<u>DNAJC12</u>	4.2	4.3E-6	DnaJ heat shock protein family (Hsp40) member C12/JDP1	
	CRELD2	3.2	0.0006	Cysteine rich with EGF like domains 2	Biochem Biophys Res Commun. 2009;387(3):504-10.
	HYOU1	3.2	0.0007	Hypoxia up-regulated 1/GRP170	Cell Struct Funct. 2008; 33(1):75-89
	PDIA4	2.7	0.008	Protein disulfide isomerase family A, member 4; chaperone activity/Erp72	Cell Rep. 2013; 3(4): 1279–1292.
	HSP90B1	2.7	0.009	heat shock protein 90 beta family member 1/GRP94	J Biol Chem. 2011; 286(52):44855-68
	HSPA5	2.6	0.01	Heat shock protein family A (Hsp70) member 5/GRP78/Bip	Mol Cell Biol. 2005; 25(11):4529-40; Mol Biol Cell. 2016; 27(9): 1536–1551
	MANF	2.4	0.02	Mesencephalic astrocyte derived neurotrophic factor/ARP	Circ Res. 2008; 103(11):1249-58.
	DNAJB11	2.5	0.02	DnaJ heat shock protein family (Hsp40) member B11/ERdj3	Cell Rep. 2013;3(4):1279-92
	HERPUD1	2.4	0.03	Homocysteine inducible ER protein with ubiquitin like domain 1	Cell Rep. 2013;3(4):1279-92.
	CRELD1	2.4	0.03	Cysteine rich with EGF like domains 1/ AVSD2; CIRRIN	Elife. 2014 ;3:e01694 Biochem Biophys Res Commun. 2009; 387(3):504-10

Disease Models & Mechanisms • Supplementary information

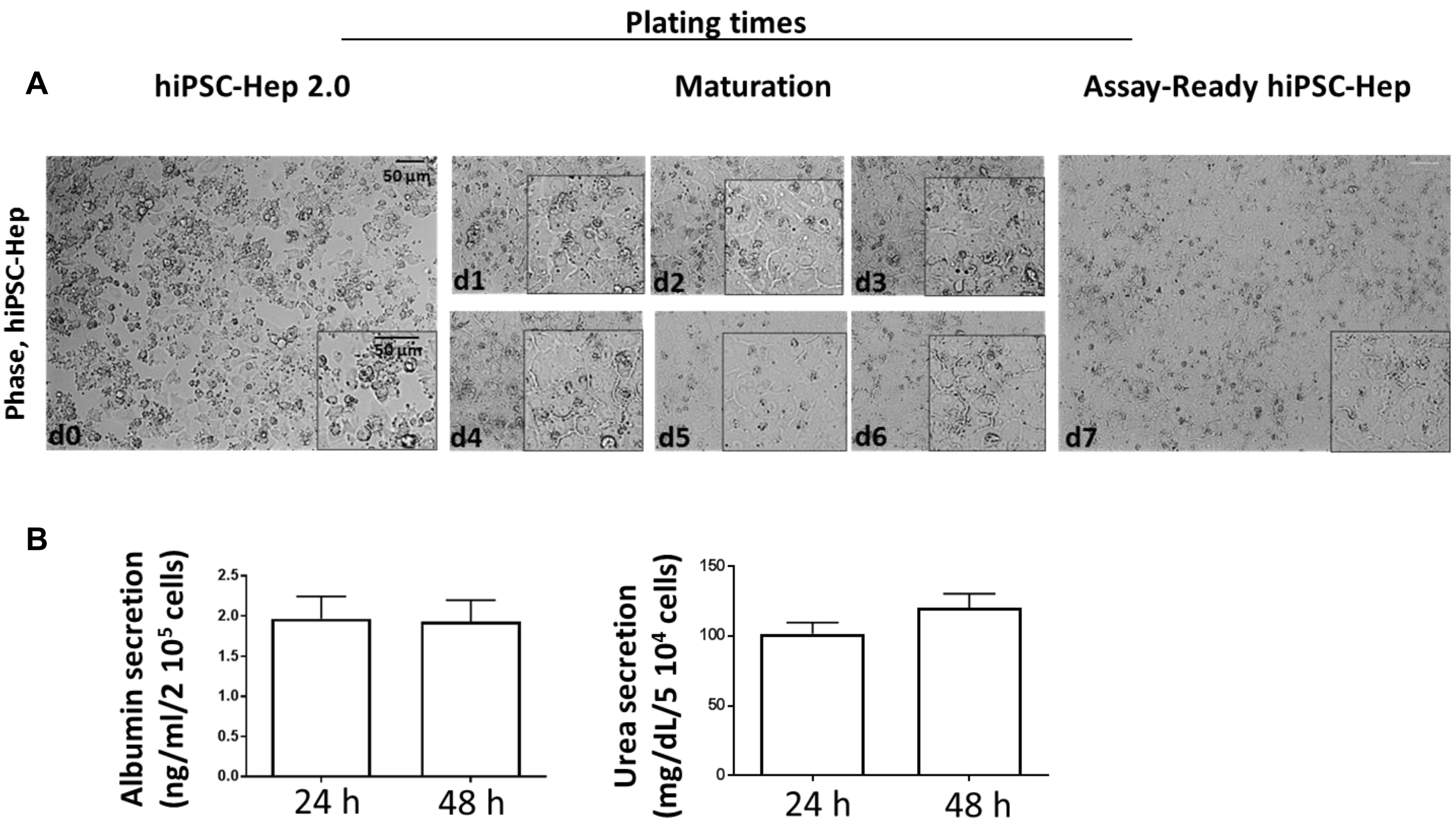

Figure S1. Morphological and functional characterization of hiPSC-Hep. (A) Representative light microscopy images of the cell morphology at different stages of maturation post thaw (day 0 to 7). (B) Albumin and urea secretion into the medium per number of cells as measured by ELISA at 24 and 48 h after day 9. Data are mean±s.d for three experimental determinations.

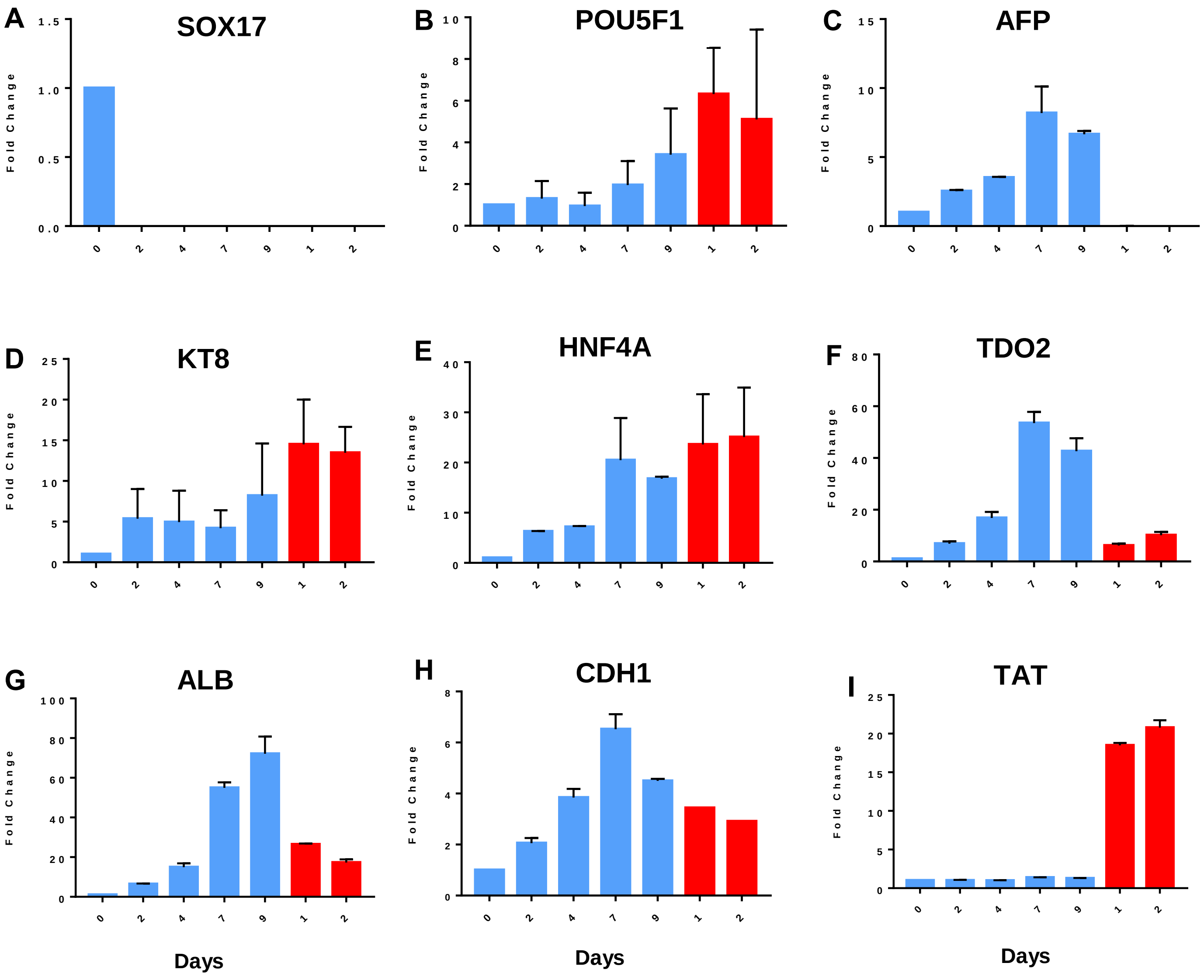

Figure S2. Expression levels of genes associated with hepatic maturation. The reference genes were selected as endogenous control genes to assess the hiPSC-Hep (blue bars) hepatic maturation status in comparison with primary human hepatocytes (red bars)**.** The x-axis represents the time (in days) in culture during hepatic maturation. The y-axis represents the fold change values from the RT-qPCR TaqMan analysis. Data are mean mean±s.d for two experimental determinations, each performed with triplicate replicates.

Disease Models & Mechanisms • Supplementary information

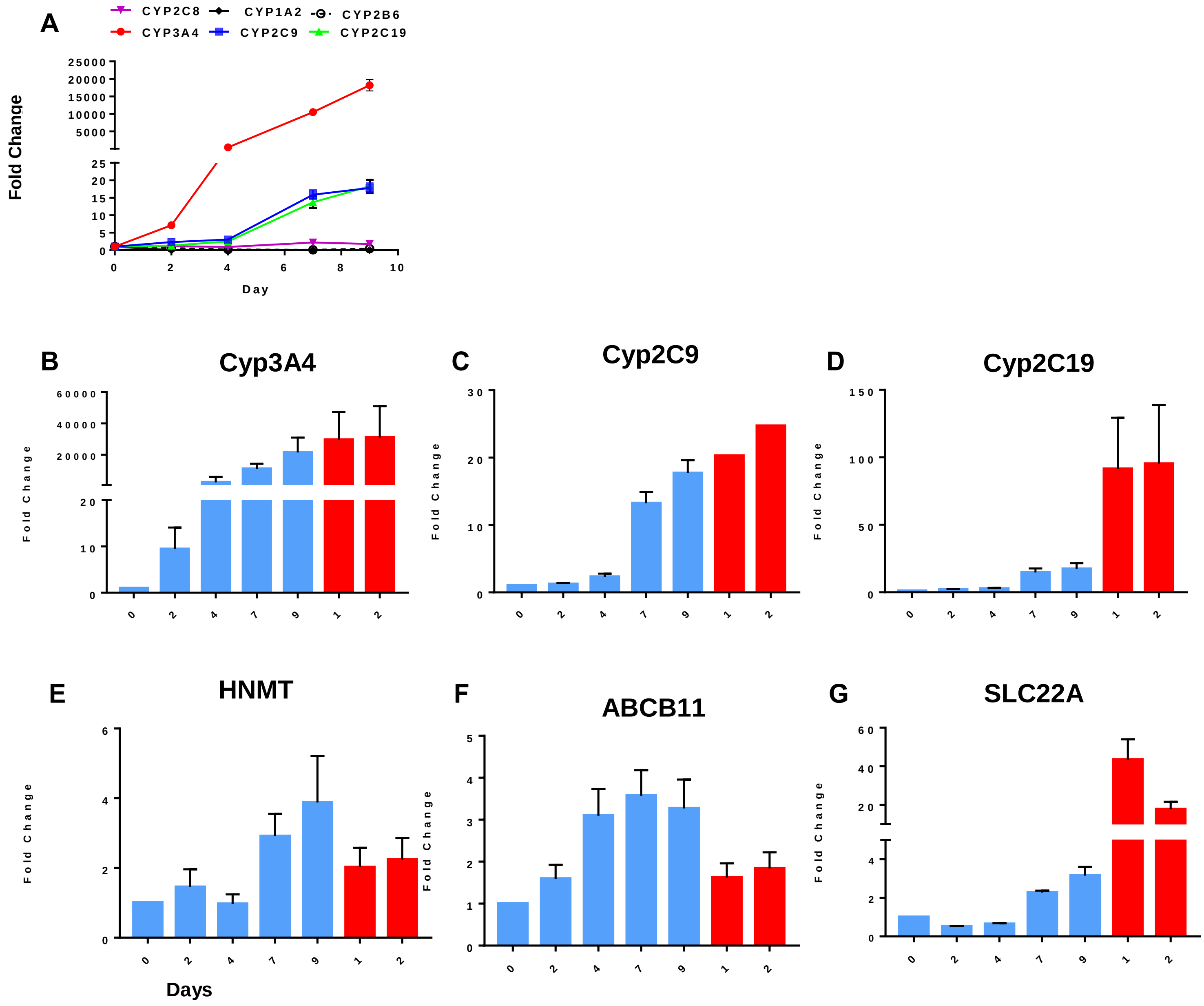

Figure S3. Time-dependent changes in gene expression for phase I, II and III metabolism genes. (A) The time course for cytochrome P450 enzymes in the hiPSC-Hep. (B-G) The reference genes were selected as endogenous control genes to hiPSC-Hep (blue bars) hepatic maturation status in comparison with primary human hepatocytes (red bars). The x-axis represents the time (in days) in culture during hepatic maturation. The y-axis represents the fold change values from the RT-qPCR TaqMan analysis. Data are mean mean±s.d for two experimental determinations, each performed with triplicate replicates.

Disease Models & Mechanisms • Supplementary information

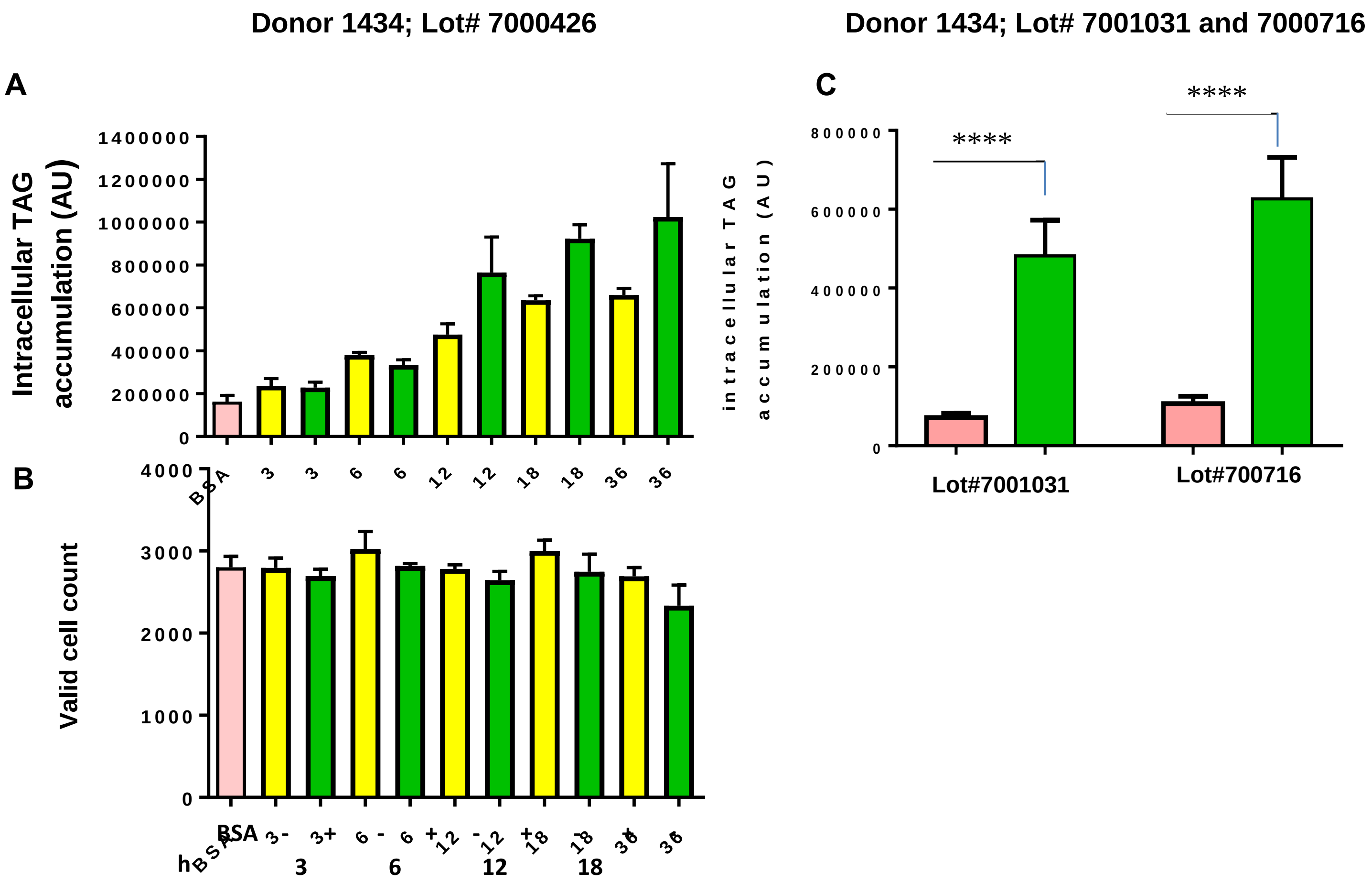

Figure S4. Lipid accumulation for unique lots of iPSC-Hep. (A) Intracellular lipid accumulates in the presence of 25 µM oleic acid and 200µM palmitic acid alone (yellow bars) or in combination with 1µM Thapsigargin (green bars) over the time course compared to BSA-treated hiPSC-Hep (pink bar) and is maximal between 12 to 36 h as determined by high-content analysis and shown in Fig 1 and described in Materials and Methods. (B) Treatments in (A) had no adverse effect on valid cell count. Data are mean±s.d for three experimental determinations, each determined from the average of three wells and seven fields per well. (C) Different lots of the hiPSC-Hep donor line 1434 shows reproducible lipid accumulation change between BSA-treated hiPSC-Hep (pink bar) and hiPSC-Hep treated with 25 µM oleic acid, 200µM palmitic acid and 1µM Thapsigargin mix (green bars) across a 384w plate. Data are mean±s.d for 32 biological determinations, each determined from the average of three wells and three fields per well. **** $P<0.0001$.

Disease Models & Mechanisms • Supplementary information

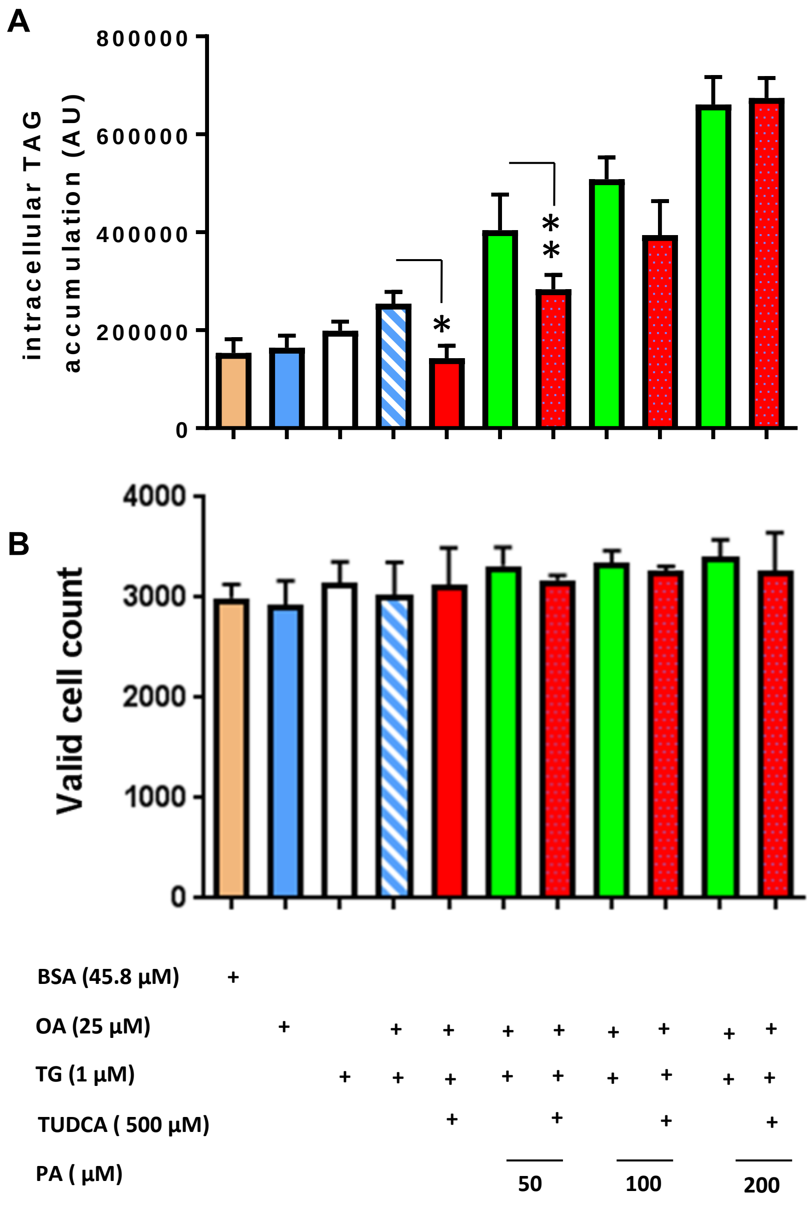

Figure S5. Exposure to TUDCA reduces lipid accumulation. (A) Lipid-uptake by the hiPSC-Hep treated with palmitic acid (50-200 µM) and 25 µM oleic acid and 1µM Thapsigargin mix in the presence (red bars) or absence (green bars) of TUDCA was assessed by Bodipy 493/503 staining. (B) Nuclei were stained with Hoechst dye (blue) for valid cell count. Data are mean±s.d for two experimental determinations, each determined from the average of three wells and seven fields per well. *$p \leq 0.03$, **$p \leq 0.009$.

Disease Models & Mechanisms • Supplementary information

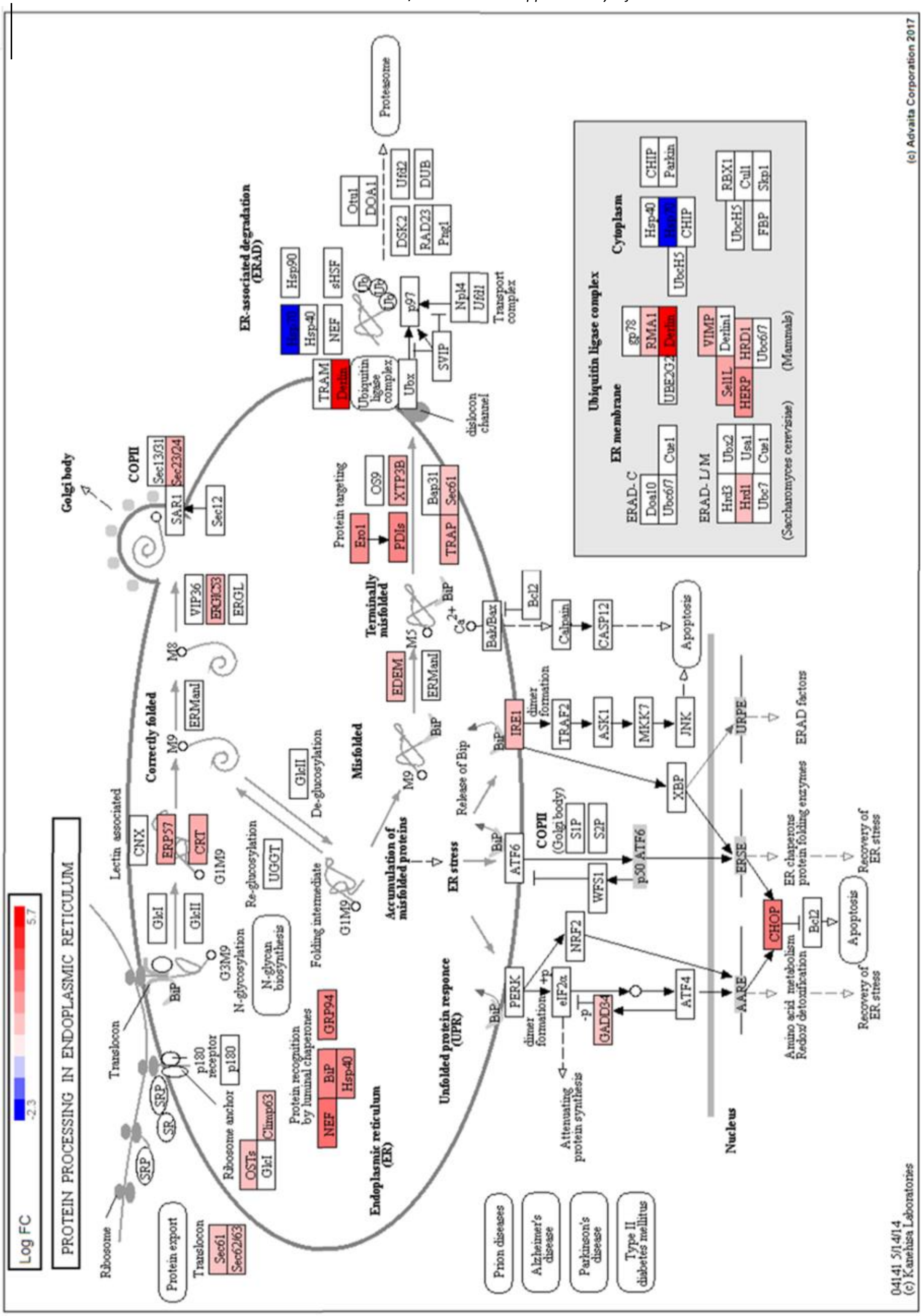
Log FC
-2.3
5.7
PROTEIN PROCESSING IN ENDOPLASMIC RETICULUM
Ribosome
Translocon
Protein export
Sec61
Sec62/63
SRP
SR
p180 receptor
p180
Ribosome anchor
OSTs
GlcI
Climp63
N-glycosylation
G3M9
N-glycan biosynthesis
Lectin associated
CNX
ERP57
CRT
GlcII
UGGT
Re-glucosylation
Folding intermediate
G1M9
Correctly folded
M9
ERManI
M8
VIP36
ERGIC53
ERGL
Golgi body
COPII
SAR1
Sec13/31
Sec23/24
Sec12
Protein recognition by luminal chaperones
NEF
BiP
Hsp40
GRP94
Endoplasmic reticulum (ER)
De-glucosylation
Misfolded
Accumulation of misfolded proteins
ER stress
Release of Bip
Terminally misfolded
EDEM
M5
Protein targeting
Ero1
PDIs
OS9
XTP3B
TRAP
Bap31
TRAM
Derlin
Ubiquitin ligase complex
Ubx
SVIP
p97
Npl4
Ufd1
Transport complex
dislocon channel
ER-associated degradation (ERAD)
Hsp70
Hsp90
sHSF
Otu1
DOA1
DSK2
Ufd2
RAD23
Png1
DUB
Proteasome
Unfolded protein responce (UPR)
PERK
eIF2α
GADD34
ATF4
NRF2
ATF6
WFS1
S1P
S2P
p50 ATF6
IRE1
TRAF2
ASK1
MKK7
JNK
XBP
Bak/Bax
Bcl2
Calpain
CASP12
Apoptosis
Attenuating protein synthesis
dimer formation
AARE
ERSE
UPRE
CHOP
Nucleus
Amino acid metabolism Redox/ detoxification
ER chaperons protein folding enzymes
ERAD factors
Recovery of ER stress
Prion diseases
Alzheimer's disease
Parkinson's disease
Type II diabetes mellitus
Ubiquitin ligase complex
ER membrane
Cytoplasm
ERAD-C
Doa10
Ubc6/7
Cue1
ERAD- L/ M
Hrd3
Hrd1
Ubx2
Usa1
Ubc7
(Saccharomyces cerevisiae)
gp78
RMA1
UBE2G2
VIMP
Derlin1
SelIL
HERP
HRD1
Ubc6/7
(Mammals)
Hsp40
CHIP
UbcH5
Parkin
RBX1
Cul1
Skp1
FBP
04141 5/14/14
(c) Kanehisa Laboratories
(c) Advaita Corporation 2017

Figure S6. Prediction of emergent biological relationships among genes and their pathways. Kyoto Encyclopedia of Genes and Genomes database (KEGG) map of the canonical protein processing in ER pathway by iPathwayGuide during induced-ER stress in hiPSC-Hep. Blue and red colors indicate low to high perturbation and each gene is highlighted in all locations it occurs in the diagram. The original Protein processing in ER pathway was obtained from the freely available Kegg Pathway Database (http://www.kegg.jp/kegg/pathway.html).

Disease Models & Mechanisms • Supplementary information

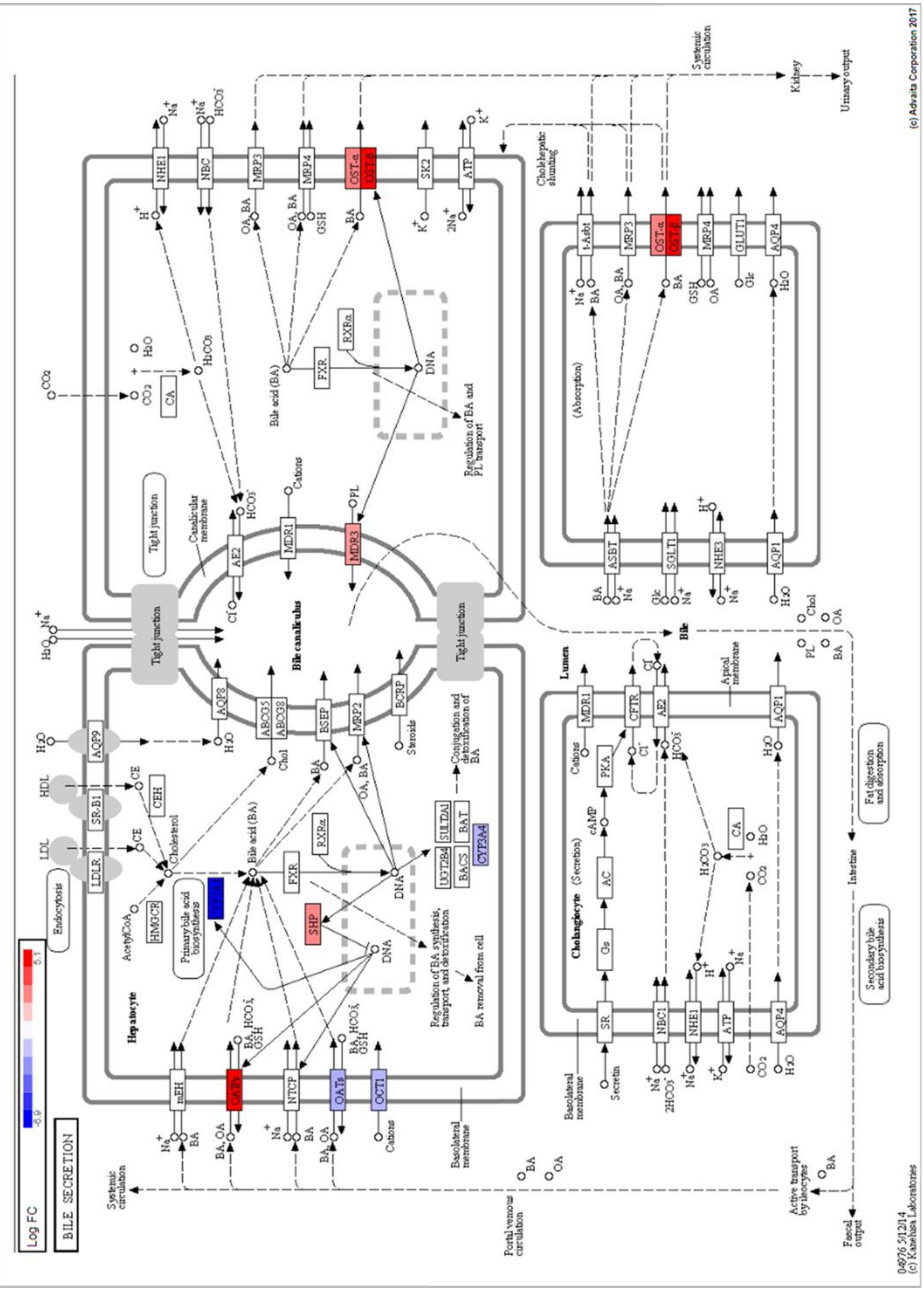
BILE SECRETION
Log FC
-8.9
5.1
Hepatocyte
Cholangiocyte
Bile canaliculus
Tight junction
Lumen
Bile
Basolateral membrane
Canalicular membrane
Apical membrane
Endocytosis
Primary bile acid biosynthesis
Secondary bile acid biosynthesis
Fat digestion and absorption
Regulation of BA synthesis, transport, and detoxification
BA removal from cell
Conjugation and detoxification of BA
Regulation of BA and PL transport
Cholehepatic shunting
Systemic circulation
Portal venous circulation
Kidney
Urinary output
Intestine
Fecal output
Active transport by ileocytes
LDL
HDL
LDLR
SR-B1
AQP9
CEH
HMGCR
Cholesterol
Bile acid (BA)
FXR
RXRα
SHP
DNA
UGT2B4
SULT2A1
BACS
BAT
CYP3A4
mEH
OATPs
NTCP
OATs
OCT1
AQP8
ABCG5
ABCG8
BSEP
MRP2
BCRP
AE2
MDR1
MDR3
NHE1
NBC
MRP3
MRP4
OST-α
OST-β
SK2
ATP
CA
t-Asbt
GLUT1
AQP4
ASBT
SGLT1
NHE3
AQP1
SR
NBC1
Gs
AC
PKA
CFTR
Secretin
cAMP
04976 5/12/14
(c) Kanehisa Laboratories
(c) Advaita Corporation 2017

Figure S7. KEGG map of the canonical Bile secretion pathway by iPathwayGuide after OCA-TG-FA treatment in hiPSC-Hep. Red and blue boxes are up- and down-regulated genes, respectively, in the data set of OCA-TG-FA vs TG-FA-treated cells group and each gene is highlighted in all locations it occurs in the diagram.

Disease Models & Mechanisms • Supplementary information
